# Presence of Parabens and Bisphenols in Food Commonly Consumed in Spain

**DOI:** 10.3390/foods10010092

**Published:** 2021-01-05

**Authors:** Yolanda Gálvez-Ontiveros, Inmaculada Moscoso-Ruiz, Lourdes Rodrigo, Margarita Aguilera, Ana Rivas, Alberto Zafra-Gómez

**Affiliations:** 1 Department of Nutrition and Food Science, University of Granada, Campus of Cartuja, 18071 Granada, Spain; yolandagalvez@ugr.es; 2 Department of Analytical Chemistry, University of Granada, Campus of Fuentenueva, 18071 Granada, Spain; innmamoscoso@correo.ugr.es (I.M.-R.); azafra@ugr.es (A.Z.-G.); 3 Department of Legal Medicine and Toxicology, University of Granada, 18071 Granada, Spain; lourdesr@ugr.es; 4 Department of Microbiology, Faculty of Pharmacy, University of Granada, Campus of Cartuja, 18071 Granada, Spain; maguiler@ugr.es

**Keywords:** parabens, bisphenols, food, Spain

## Abstract

Given the widespread use of bisphenols and parabens in consumer products, the assessment of their intake is crucial and represents the first step towards the assessment of the potential risks that these compounds may pose to human health. In the present study, a total of 98 samples of food items commonly consumed by the Spanish population were collected from different national supermarkets and grocery stores for the determination of parabens and bisphenols. Our analysis demonstrated that 56 of the 98 food samples contained detectable levels of parabens with limits of quantification (LOQ) between 0.4 and 0.9 ng g^−1^. The total concentration of parabens (sum of four parabens: ∑parabens) ranged from below the LOQ to 281.7 ng g^−1^, with a mean value of 73.86 ng g^−1^. A total of 52% of the samples showed detectable concentrations of bisphenols. Bisphenol A (BPA) was the most frequently detected bisphenol in the food samples analysed, followed by bisphenol S (BPS) and bisphenol E (BPE). Bisphenol AF (BPAF), bisphenol B (BPB) and bisphenol P (BPP) were not found in any of the analysed samples. LOQ for these bisphenols were between 0.4 and 4.0 ng g^−1^.

## 1. Introduction

The antimicrobial activity of parabens has been known since 1924 and for this reason these alkyl esters of *p*-hydroxybenzoic acid have been extensively used as preservatives in many consumer products such as health and personal care product and foodstuffs [1,2,3]. Parabens are regulated as preservatives in Commission Regulation (EU) No 1004/2014 on cosmetic products that sets a maximum limit of 0.4% and 0.8% for single esters and mixtures of esters, respectively [4]. 

There are growing concerns about the presence of these preservatives in pharmaceuticals and cosmetic products associated with their estrogenic effects demonstrated by in vivo and in vitro studies [5]. This disrupting hormone activity seems to be linked to the length of the alkyl chain, with long-chain parabens like propyl 4-hydroxybenzoate (PropPB) and butyl 4-hydroxybenzoate (ButPB) being those of highest concern [6]. In 2010, the EU Scientific Committee on Consumer Safety (SCCS) considered that the use of methyl 4-hydroxybenzoate (MetPB) and ethyl 4-hydroxybenzoate (EthPB) at the maximum authorized concentrations is safe but due to the lack of scientific data, the Committee cannot ascertain that the use of PropPB and ButPB at the maximum concentrations is completely safe [7]. With respect to the use of MetPB and EthPB (food additives E218 and E214, respectively) and their sodium salts (E219 and E215, respectively) in foodstuffs, the maximum permitted levels (MPLs) are between 300 and 1000 mg kg^−1^. No other parabens are approved for use in food. The European Food Safety Authority (EFSA) concluded that an ADI (Acceptable Daily Intake) of 0–10 mg kg^−1^ body weight (bw) day^−1^ could be set for MetPB and EthPB and their sodium salts [8]. In 2015, the European Medicines Agency (EMA) reported evidence of adverse health effects related to the intake of PropPB and ADI at 1.25 mg kg^−1^ bw day^−1^ [9]. Despite having been used as food preservatives for many decades, little information is available about parabens concentration in certain foods and dietary exposure [10,11].

Bisphenol A (BPA) is also known to disrupt hormone function. This chemical is produced in large volumes and it is used primarily to harden polycarbonate plastics and epoxy resins [12] used in a wide variety of consumer products including beverage bottles, food can coatings, plastic tableware, thermal paper, and medical devices. The most common route of human exposure is food and beverage consumption [13]. The harmful effects on the reproductive, cardiovascular, immune and metabolic systems related to human exposure to BPA have been extensively described [14]. In 2017, BPA was included in the European Chemical Agency (ECHA) Candidate List of substances of very high concern. In view of the recent limitations on the use of BPA in food contact materials [15,16,17,18], the food packaging industry is exploring alternatives to replace BPA in these materials [19,20,21].

In this regard, BPA substitutes such as BPS [4,4′-sulfonyldiphenol], BPF [4,4′-dihydroxydiphenylmethane], BPB [2,2-bis(4-hydroxyphenyl) butane], BPE [1,1-Bis (4-hydroxyphenyl) ethane], and BPAF [4,4′-(hexafluoroisopropylidene) diphenol] are being used as alternatives to BPA in some industrial applications for manufacturing polycarbonate resins [22,23,24,25,26]. As with BPA, these replacement chemicals have a structure similar to BPA and therefore also exhibit endocrine disrupting properties [19,27,28]. However, studies on the occurrence of bisphenols, other than BPA, in foodstuffs are limited. In 2015, the EFSA re-examined BPA exposure and toxicity issues, reducing the BPA tolerable dietary intake (TDI), previously set at 50 μg kg^−1^ bw day^−1^ [18], to 4 μg kg^−1^ bw day^−1^ [29]. No specific limits were indicated for other types of bisphenols. 

In children, the food chain is the main exposure route to parabens and bisphenols. In addition, children are especially vulnerable to developmental exposure and it has been reported that exposure levels in infants and children in relation to their body weight are higher than in adults [30]. Recently, we have developed an analytical method to determine BPB, BPS, BPE and BPP concentration in food products for children [19]. 

We conducted an exhaustive literature review that showed that the analytical techniques most commonly used for the extraction of the analytes included in the present study are solid phase extraction (SPE) and QuEChERs (Quick, Easy, Cheap, Effective, Rugged, and Safe), and QuEChERs and liquid–liquid extraction (LLE) for the clean-up phase. The main separation techniques are liquid chromatography and gas chromatography coupled with mass spectrometry, used for analyte detection. Other studies have used an alternative method that uses liquid chromatography coupled with fluorescence detection (data available at “Supplementary Material”, Tables S1 and S2).

Bisphenols have been the focus of extensive research over the last years; however the available studies are mainly focused on BPA. Since BPA and its substitutes show similar endocrine disrupting properties and effects, further study of these compounds used to replace BPA is warranted. In this work, the concentration of parabens and bisphenols in 98 samples of food items commonly consumed by Spanish population is determined, which are collected from different supermarkets and grocery stores. Although several studies have addressed the presence of bisphenols in food items in Spain, they are very limited regarding the number of samples of food and food packaging analysed. In addition, there are very few studies on the presence of parabens in food and to our knowledge, no studies have been conducted on paraben concentrations in such a wide range of food products commonly consumed by the Spanish population. Moreover, existing studies focus on one class of endocrine disruptors only and none of them include the analysis of both bisphenols and parabens.

## 2. Material and Methods

### 2.1. Chemicals

**Figure 1:**
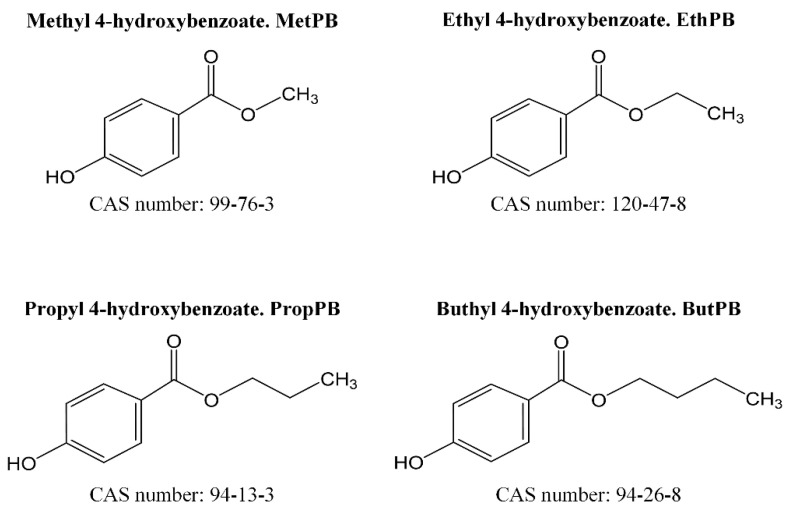
Parabens analysed in the present work.

**Figure 2:**
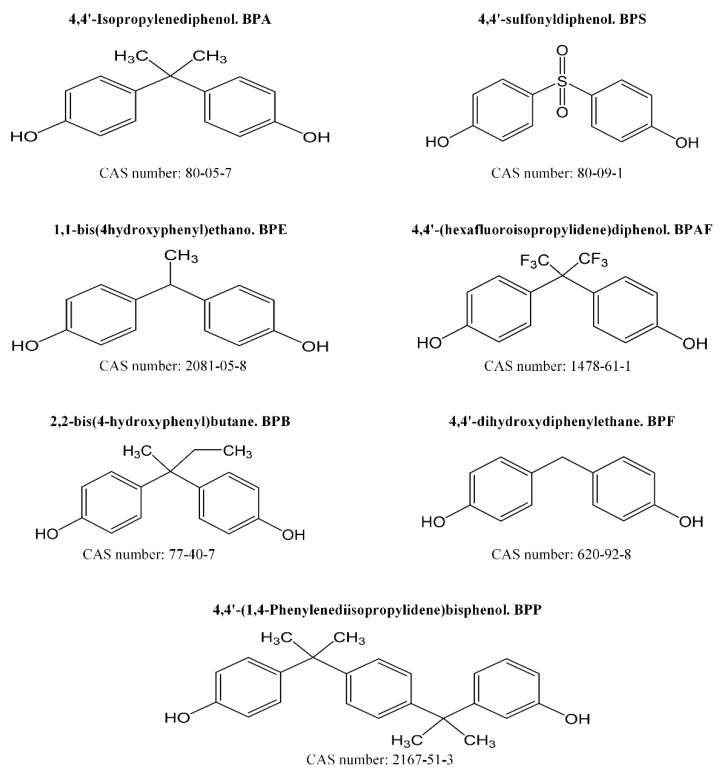
Bisphenols analysed in the present work.

All reagents were of analytical grade. Ultrapure water (18.2 MΩ-cm) was prepared with the in-house Milli-Q Plus^®^ system from Merck Millipore. MetPB, EthPB, PropPB and ButPB (≥99% purity) were supplied by Alfa Aesar (Thermo Fisher Scientific, Kandel, Germany) (Figure 1). BPA, BPF, BPS, BPAF, BPP (≥99% purity), BPE, BPB (≥98% purity), and deuterium labelled bisphenol A (BPA-d_16_, ≥99% purity) were supplied by Sigma-Aldrich (Madrid, Spain) (Figure 2). Ethyl-d_5_-paraben (EthPB-d_5_) was obtained from Toronto Research Chemicals (Toronto Research Chemicals, NY, Canada). Stock solutions of bisphenols (100 mg L^−1^), parabens (100 mg L^−1^) and internal standards BPA-d_16_ and EthPB-d_5_ (10 mg L^−1^) were prepared in methanol (MeOH). The working standard solution was prepared by diluting the stock solutions of the 11 analytes investigated (10 mg L^−1^) with MeOH and was stored in amber glass vials at −20 °C until analysis. Calibration standards were prepared by spiking food matrix with working standard solution. Liquid chromatography-mass spectrometry (LC-MS) solvents methanol and acetonitrile were provided by VWR Chemicals (VWR international, Barcelona, Spain). Ammonia solution 25% for LC-MS used as mobile phase modifier and sodium hydroxide (NaOH) pellets of reagent grade (≥98% purity) were from Sigma-Aldrich (Madrid, Spain). Sodium chloride (NaCl) and magnesium sulphate (MgSO_₄_) were from Panreac (Barcelona, Spain). Sorbents, silica functionalized with octadecyl groups (C18) and primary-secondary amine (PSA) were from Scharlab (Barcelona, Spain).

### 2.2. Instrumentation and Software

A Waters Acquity Ultra-high Performance Liquid Chromatography™ I-Class system (Waters Corporation, Milford, CT, USA) was used for the determination of seven bisphenols and four parabens. A Waters Xevo^®^ TQ-XS (Waters Corporation, Milford, CT, USA) with an orthogonal Z-Spray™ electrospray ionization (ESI) source (Waters Corporation, Milford, CT, USA) was used for the spectrometric measurements. The column was a Waters UPLC^®^ BEH C_18_ (2.1 mm × 50 mm, 1.7 μm particle size). A ScanVac CoolSafe™ lyophilizer (Lynge, Denmark) was used for lyophilization of food samples. Other laboratory equipment was a vortex-mixer (IKA, Staufen, Germany), a GX400 laboratory balance (Mettler-Toledo, Columbus, OH, USA), a Universal 32 centrifuge (Hettich, Tuttlingen, Germany), a Spectrafuge™ 24D centrifuge (Labnet International, Inc., Edison, NJ, USA) and a SBHC0NC sample concentrator (Stuart, Staffordshire, UK), and an Ultrasons-HD series ultrasonic bath (Selecta, Barcelona, Spain). For the treatment and analysis of data and for equipment control, MassLynx 4.1 software (Waters Corporation, Milford, CT, USA) from Waters was used.

### 2.3. Food Sampling

The food items included were selected among the most consumed items by the Spanish population. Most of the selected food was packaged in plastic, cans, paper trays, paperboard, foil and carton packages. The food items included in this study were collected from different national supermarkets and grocery stores and were segregated into the categories defined by the NOVA food classification system based on their nature, extent and purpose of the industrial processes they undergo (unprocessed or minimally processed foods, processed food and ultra-processed foods) [31]. A singular feature of the NOVA classification is the definition of ultra-processed food, which are not modified foods, but formulations obtained by the processing of cheap industrial ingredients that usually also include additives to make them more durable and tastier [31].

### 2.4. Analytical Methods

Solid foods and dairy products were lyophilized prior to the treatment of the samples and their subsequent analysis. For sample treatment the method developed by García-Córcoles et al. (2018) [19] with some modifications was used. Briefly, 2 g of each sample were weighed into a 10 mL glass tube and 5 μL of a solution of internal standards in MeOH, BPA-d₁₆ and EthPB-d_5_ (10 ppm) was added. Samples were homogenized in 2 mL ultrapure water and 6 mL acetonitrile in a vortex-mixer for one minute, and subsequently bath sonicated for 30 min at 15 °C. NaCl 1 g was added to each food sample and centrifuged for 5 min at 4000 rpm (2594× *g*). The upper organic layer was transferred to a 10 mL glass tube and 600 mg MgSO₄ and 150 mg PSA were added to remove proteins, carbohydrates, and lipids. The mixture was stirred in a vortex-mixer for one minute and centrifuged for 5 min at 4000 rpm (2594× *g*). The supernatant was evaporated to dryness into a centrifugal evaporator (2000 rpm, 50 °C). For the chromagraphic analysis a new method based on ultrahigh performance liquid chromatography-tandem mass spectrometry was optimized. The extracts were reconstituted by adding 250 μL of a MeOH/H_2_O mixture (50:50, *v/v*) and stirring in a vortex mixer until homogenization. After centrifugation for 5 min at 4000 rpm (2594× *g*), the extract obtained was transferred to a glass vial and directly injected into the ultra-high performance liquid chromatography-tandem mass spectrometric (UHPLC-MS/MS) system.

### 2.5. Ultrahigh Performance Liquid Chromatography—Tandem Mass Spectrometry Analysis

The chromatographic conditions optimized for the analysis are summarized next. An Acquity UPLC^®^ BEH C18 column (2.1 mm × 50 mm, 1.7 μm particle size) was used for analyte separation at 40 °C column temperature. Based on our previous experience, 0.025% (*v/v*) ammonium in water (solvent A) and MeOH (solvent B) were used as mobile phase [32]. Flow rate was 0.35 mL min^−1^ and injection volume was 10 μL. The mobile phase gradient in the food samples was: 0.0 to 1.0 min, 10% solvent B; 1.0 to 6.0 min, 10 to 90% B; 6.0 to 6.1 min, 90 to 100% B; 6.1 to 6.6 min back to 10% B (run time 10 min). For sensitive and selective quantification of the target compounds in food samples with the highest specificity and sensitivity, negative electrospray ionization in multiple-reaction-monitoring (MRM) mode was used. The most abundant transition monitored was used for quantification and the second to confirm identification. In two cases (BPA and BPE) only one transition was sensitive and useful. Table 1 shows the optimized parameters for the UHPLC-MS/MS analysis.

### 2.6. Method Validation

Validation and quality parameters of the method, selectivity, linearity, and sensitivity (limit of detection (LOD) and limit of quantification (LOQ)) and accuracy (precision and trueness), were evaluated for method validation. A non-contaminated sample was used as a blank to verify method selectivity, and after spiking to confirm a good sensitivity and accuracy.

**Figure 3:**
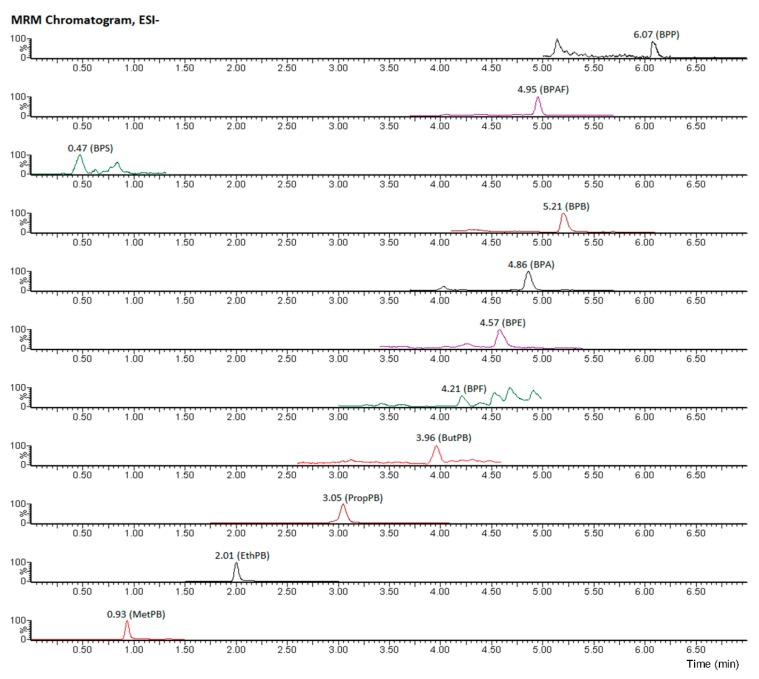
Chromatogram of the lower calibration level.

To minimize the influence of the matrix, quantitative analyses were performed based on matrix-matched calibration curves. The calibration curves were obtained from the analyses of each analyte in blank samples spiked with different concentrations of the analytes: 0 ng g^−1^, 1 ng g^−1^, 5 ng g^−1^, 10 ng g^−1^, 25 ng g^−1^, 50 ng g^−1^, 100 ng g^−1^ and 250 ng g^−1^. Figure 3 shows a chromatogram of a standard (1 ng g^−1^).

Method selectivity was determined by analysing the signal of the blanks and verifying the absence of peaks in the retention times of the target analytes (Figure S1). LODs and LOQs were defined as the analyte concentration producing an analytical signal of three (LOD) and ten (LOQ) times the signal-to-noise ratio. Table 2 shows the parameters evaluated for calibration, LODs, LOQs and linearity for each analyte. Finally, method accuracy in terms of precision and trueness was evaluated by spiking blank samples at three concentration levels (1, 100, 250 ng g^−1^) of each compound investigated. Three replicates per day in six different days were obtained. Recovery data (trueness confirmation) were between 91% and 106% for all the analytes, with a standard relative deviation (precision confirmation) lower than 12% in all cases.

### 2.7. Estimation of Dietary Exposure in Spanish Children 

Dietary intakes of parabens and bisphenols were calculated for children aged 6–9 years. This age group is one of the most vulnerable groups to endocrine disrupting chemicals and extensive information about their food consumption, dietary habits and anthropometric measures is available from different surveys conducted by the Spanish Agency for Food Safety and Nutrition (*AESAN*) in collaboration with European Agencies such as EFSA.

Total daily exposure was calculated by multiplying the daily intake of food groups (g day^−1^) by the mean bisphenol or paraben concentrations (μg kg^−1^) and dividing this value by body weight in kg. The daily intake of food groups was obtained from the ENALIA study, a cross-sectional survey conducted under the umbrella of *AESAN* and EFSA on a nationally-representative sample of children and adolescents aimed at collecting data on food consumption. [33]. The bisphenol and paraben mean concentrations used were those calculated separating the analysed foods into the categories defined by ENALIA study (meat and derivatives, fish and derivatives, cereals and derivatives, vegetables and derivatives, fruits and derivatives, dairy and derivatives, eggs, salty snacks and pre-cooked foods) (https://www.aesan.gob.es/AECOSAN/web/seguridadalimentaria/subdetalle/enalia.htm#4).

The body weight used for the calculations of total daily exposure was estimated at 29.8 kg based on the data provided by a survey on weight and height conducted on a nationally-representative sample of Spanish children aged 6–9 years [34].

### 2.8. Statistics

SPSS v.23 (version 23, IBM^®^ SPSS^®^ Statistics, Armonk, NY, USA) and Statgraphics plus 5.0 (version 5, Statpoint Technologies Inc., Warrenton, VA, USA) packages were used for the statistical treatment of analytical data. For calculations, the non-detected and non-quantified values/compounds were excluded from data treatment. The strength of the association between bisphenol and paraben concentration in food samples was measured by Spearman’s correlation. A *p* < 0.05 value was considered significant. 

## 3. Results and Discussion

### 3.1. Parabens in Food Samples

Our results found detectable levels of parabens in 56 out of the 98 food samples analysed. The most frequently detected paraben was MetPB (49%), followed by EthPB (15.5%), and PropPB (10.2%) (Table 3). The detection frequency of ButPB was lower than for the rest of parabens (8.1%) (Table 3). This frequency pattern found in our food samples is consistent with the fact that generally the lower esters are the are the preferred parabens in foods [35] and is similar to that reported in human urine, blood and breast milk from the US population, where the most frequently detected paraben is MetPB [36].

The total concentration of parabens (∑parabens) ranged from below the LOQ to 281.7 ng g^−1^, with a mean value of 73.86 ng g^−1^ (Table 4, Table 5 and Table 6). Some of the samples such as chicken burger (281.7 ng g^−1^), frozen chopped onion (231.9 ng g^−1^), eggs (229.9 ng g^−1^), and milk bread with chocolate (145.3 ng g^−1^), contained remarkably high concentrations of MetPB. The highest concentration of EthPB was found in canned tuna in oil (146.9 ng g^−1^) and in anchovy stuffed olives (86.9 ng g^−1^), and of PropPB in milk bread with chocolate (145.3 ng g^−1^) and in olives (85.2 ng g^−1^). ButPB was found in lower concentrations than the other parabens, with its highest levels found in pineapple in plastic packaging (68.9 ng g^−1^). An example of a chromatogram of one of the food samples analysed in this study is shown in Figure S2.

The literature review revealed only a small number of studies about the presence of parabens in food, maybe related to the fact that the source of parabens in food is not known with certainty (use as antimicrobial agents and in food packaging) [2,3,37]. We compared the concentrations of parabens detected in this study with those reported in other studies carried out in the European Union (EU) (Table S1) as the levels should comply with EU legislation on food safety assessment. Moreover, a LOD from 0.1 to 0.3 ng g^−1^ and a LOQ from 0.4 to 0.9 ng g^−1^ found for the studied analytes mean that this method can be consider sensitive as other authors have reported similar values for parabens (information available at “Supplementary Material”, Table S1).

The concentration of parabens found in this study is higher than that found in other works (Table S1). A possible explanation for this is that the foods analysed in this study are different and come in different packages than those analysed in previous works, which makes comparison difficult. In addition, food categorization into specific groups is vague/unclear/unspecific in previous works. Lastly, most of the foods selected for the present study are processed foods, where parabens are extensively used as additives, and come in plastic packaging, from where parabens may leach into the food inside, hence the higher concentrations found. The concentrations of parabens varied widely even within the same category of foodstuff, and processed foods generally contained higher paraben concentrations than unprocessed/fresh foods [2,3].

As in this study, the most frequently detected paraben in European studies was MetPB, which is also the one detected in the highest concentrations in food. MetPB concentrations previously reported in European studies were similar to the concentrations found in our food samples, ranging from below the LOD to 84.69 ng g^−1^ [38,39,40,41,42,43,44]. The European studies have also reported that other parabens frequently detected were EthPB (ranging from <LOD to 0.82 ng g^−1^) and PropPB (ranging from <LOD to 7.43 ng g^−1^) [38,40,41,42,43,44].

On the other hand, ButPB was not detected in the European studies (<LOD) [41,42], but we found detectable concentrations of this paraben (ranging from <LOD to 145.3 ng g^−1^) although with the lowest frequency (8%) in the food samples analysed (Table 3).

Isopropylparaben and benzylparaben have been detected in European studies in food samples [38,40,41,43,44,45], but these parabens have not been analysed in the present study; therefore, they should be included in future studies for their determination in food. 

Pearson’s correlations revealed that EthPB concentration was positively associated with PropPB (*p* = 0.0025; Spearman’s coefficient 0.335) and ButPB (*p* = 0.0001; Spearman’s coefficient 0.506) concentrations. These correlations found in the food samples analysed show that EthPB and PropPB originate from the same sources. However, the source of parabens in foods is not completely understood but their use as broad-spectrum antimicrobial preservatives used in processed foods may be a potential source [2,3] as well as the use of certain food packaging materials where parabens are added as antimicrobials from where they can be released into the food inside [46,47,48]. We found no differences in the concentrations and frequency of paraben occurrence among the food in different packaging (Table 4, Table 5 and Table 6). We also found detectable concentrations of MetPB in two different brands of chocolate milkshake samples packed in carton. However, in a recent study conducted in Spain [49] reported MetPB concentrations ranging from nonquantifiable to 155.359 ng g^−1^ in milk carton samples. 

Parabens were also detected in eggs, which could be explained by the ingestion of paraben-contaminated feed or soil that penetrate into chicken tissues and are subsequently transferred into eggs [50]. Parabens were also detected in non-packaged fruit and vegetables as parabens may naturally occur in some fruits and vegetables and may contribute to disease resistance through their antimicrobial and antifungal properties [51,52,53]. Furthermore, EthPB has been reported to have allelopathic functions [54,55] and MetPB has been also found in a wide variety of plant species and it could be applied in the preparation of bio-based poly (ether ester) materials [56].

### 3.2. Bisphenols in Food Samples

Diet accounts for up to 99% of BPA exposure [57]. Table 4, Table 5 and Table 6 show the concentrations of bisphenol analogues and the sum of their concentrations in the food samples analysed. A total of 52% of the samples showed detectable concentrations of bisphenols. BPA was the most frequently detected bisphenol in ultra-processed food (mean = 43.28 ng g^−1^ fresh weight). BPS was the second most frequently detected bisphenol in the food samples (26.5%). BPE was found in 4.1% of food samples. However, BPF, BPAF, BPB and BPP were not found in any of the samples analysed. 

The concentration of ∑bisphenols ranged from below LOQ to 409 ng g^−1^. The highest ∑bisphenols were found in processed food, in canned tuna samples, with a mean value of 409 ng g^−1^ of BPA and 187.8 ng g^−1^ of BPS. A special concern is that canned and raw tuna is one of the most consumed fish products [25]. Moreover, bisphenol bioaccessibility is higher in canned seafood than in other food matrices with values ranging from 80 to 99% [25,58]. These results are consistent with other studies reporting higher concentrations of individual and total bisphenols in canned food than in foods sold in glass, paper, or plastic containers [21,59]. In an EFSA comprehensive report regarding the levels of BPA in foodstuff in the EU, the ratio of BPA concentrations between canned and non-canned foods ranged from 3 to 500 times, meaning that the contamination could also occur during food processing and manufacture [60].

This might be due to the leaching of bisphenols from the epoxy resins that line the cans into the food. The highest level of bisphenols (132.10 ng g^−1^ BPS) in the processed food group was found in potato chips. High concentrations of BPS were also found in pineapple samples sold in plastic packaging (44.3 ng g^−1^ BPS) included in the unprocessed or minimally processed food categories. The lowest bisphenol concentrations were found in frozen chopped garlic (mean = 1.36 ng g^−1^ BPS), cake (mean = 1.7 ng g^−1^ BPS) and burger bun (mean = 1.36 ng g^−1^ BPA). LODs and LOQs for these compounds are in the range of 0.1 to 1 ng g^−1^ and 0.4 to 4.0 ng g^−1^ respectively, which is in agreement with the results reported by similar works (information available at “Supplementary Material”, Table S2).

However, relevant bisphenol concentrations were also found in nonpackaged food, which could be explained by the potential contamination during primary production activities [61,62]. In addition, the ubiquity of plastics could also be related to the unexpected presence of bisphenols in food [63].

In recent decades the harmful effects on human health related the use of BPA in food packaging have raised much controversy [64,65], with a large number of studies dealing with BPA determination in food [45,66,67,68]. More recently, there has also been an increase in research that focuses not only on BPA but also on BPA analogues, as they have been reported to exhibit similar adverse health effects to BPA [27,69,70]. 

There is extensive literature available about BPA levels in food, but the studies assess different types of food and different BPA analogues. For this reason, comparison between studies conducted in different countries can be difficult. We performed a literature review and selected those studies conducted in the EU that determined the presence of BPA and BPA analogues in food samples (Table S2). Large variations in bisphenol concentrations were found in the present study, similar to those reported in previous studies due to methodological differences [21,71,72,73]. The results published in the European literature show concentrations ranging from <LOD to 835 ng g^−1^ (Table S2).

BPA is the most frequently detected bisphenol in the analysed food (Table S2), as in the present study (28.6%) (Table 3). Other bisphenols frequently detected in European studies were BPF (<LOD to 139.26 ng g^−1^) [20,25,26,59,74,75,76,77], followed by BPB (<LOD to 183.20 ng g^−1^) [25,63,71,76,78,79,80]. However, BPAF, BPS and BPE have a lower detection frequency in the food samples analysed from EU countries [25,26,59,63,71,77]. In contrast, in our work BPS and BPE were detected in 26.5% and 4.1% of the food samples analysed, respectively (Table 3). González et al. (2020) found BPE in two of 40 samples analysed [63], which could be explained by the use of BPE as a replacement for BPA in many products as a result of recent regulation limiting the presence of BPA. BPS is one of the most widely BPA substitutes used in the manufacturing of polycarbonate plastics and epoxy resins [81]. In addition, BPS has been frequently found in human biological samples, with detection rates of 81% [21], 65% and 30% [82,83], 70% [84], 40% [85], and 78% [86] in urine and 3% in breast milk [87]. Lastly, BPS has shown endocrine disrupting activity similar to BPA in in vivo and in vitro assays [88]. 

Other BPA analogues that have been analysed in EU studies are bisphenol AP (BPAP), bisphenol Z (BPZ), and bisphenol M (BPM), but their presence in foods is scarce [25,59,78]. In the present study, these bisphenols were not determined in our food samples, but given their reported estrogenic activity, it will be interesting to include them in future analyses.

In the present study, the association between BPA and BPS concentrations was measured with the Spearman’s correlation and a significant correlation was found between the two bisphenols in the analysed food samples (r = 0.825, *p* < 0.043). These results suggest that BPS is one of the main BPA analogues used in food-contact material and that is used together with BPA in different food-contact materials.

We found no marked differences between the concentrations of bisphenols in food packed in different materials, but the concentration of bisphenols was higherin canned tuna in oil. In contrast, in other canned foodstuff such as anchovy stuffed olives and tuna dumplings no detectable levels of bisphenols were found. Surprisingly, only detectable concentrations of BPE were found in food samples in carton packages. In contrast, a recent study conducted in Spain [49] found BPA in milk cartons at concentrations ranging from 0.0018 to 0.059 ng g^−1^. It is surprising that the inner surface of carton packages is made of four layers of low-density polyethylene. More studies should be conducted to describe the presence of bisphenols other than BPA in this kind of packaging material. BPA was also detected in non-packed apples and pears, which that could be explained by contamination during the primary production [63].

Some of the analysed samples (pineapple, canned tuna in oil, yogurt and chips) exceeded the migration limit for BPA recently established by the European Commission at 50 µg kg^−1^ [18]. However, this does not represent a health risk these food products are not consumed in excess in Spain [89]. Nonetheless, migration of bisphenols should be explored in other brands of these products. 

### 3.3. Estimated Dietary Intake in Children 

Tables S3 and S4 show the estimated dietary bisphenol and paraben intake in children aged 6–9 years for each foodstuff category.

The estimated intake of ∑PBs was 2.28 µg kg^−1^ bw day¯^1^ and 1.25 µg kg^−1^ bw day^−1^ for ∑BPs, which were higher than those calculated by Liao et al. [2,21] for United States children. However, these estimated intake values did not exceed the limit of 10 mg kg^−1^ bw day¯^1^ and 4 mg kg^−1^ bw day¯^1^ set by the EFSA for parabens and bisphenols, respectively [8,29]. The evaluation of other BPA substitutes and their TDI values was not possible because no limits have been set yet by international organizations.

This study has some limitations including the fact that several food items were not included and that the foods analysed were mainly packaged in plastic containers, hence higher concentrations of parabens and bisphenols, and therefore higher estimated daily intakes, are expected. Lastly, even though the estimated dietary intake of parabens and bisphenols are below the TDI, other routes of exposure such as household dust, air, and dental fillings must be considered. Lastly, the cumulative effect of parabens and bisphenols together with other endocrine disruptors present in food such as heavy metals, pesticides, and polybrominated diphenyl ethers could pose a risk to human health and should be studied.

## 4. Conclusions

Our findings confirm significant amounts of parabens and bisphenols detected in daily consumed products by the Spanish population. MetPB was the most frequently detected paraben in the analysed samples. Although BPA is being gradually replaced by its analogues in many food-contact materials, it is still the most frequently bisphenol detected in food, followed by BPS. The estimated dietary exposure to bisphenols and parabens did not exceed the TDIs established by the EFSA. However, because other bisphenols in addition to BPA are found in foods, a risk assessment of their presence and the establishment of limits such as TDIs for each bisphenol individually and for the sum of bisphenols are necessary. This study shows the importance of collecting more data on the occurrence of parabens and bisphenols in food to assess dietary exposure and possible health impact, especially for the more vulnerable populations.

## Figures and Tables

**Table 1:** Optimized parameters for the Ultra-high performance liquid chromatography-tandem mass spectrometry (UHPLC-MS/MS) analysis of compounds.

	t_R_ (min)	Transitions	CV	CE		t_R_ (min)	Transitions	CV	CE
BPS	0.5	249.1 → 107.5 ^b^	−4	−26	MetPB	0.9	151.0 → 91.4 ^a^	−12	−18
		249.1 → 155.5 ^a^	−4	−20			151.0 → 135.5 ^b^	−12	−14
BPF	4.2	199.1 → 76.4 ^b^	−14	−24	EthPB	2	165.1→ 91.6 ^a^	−14	−22
		199.1 → 92.4 ^a^	−14	−20			165.1 → 136.3 ^b^	−14	−14
BPE	4.6	213.1 → 197.7	−46	−18	PropPB	3	179.1 → 91.5 ^a^	−26	−22
							179.1 → 114.5 ^b^	−26	−16
BPA	4.9	227.2 → 132.9	−50	−26	ButPB	3.9	193.1 → 91.5 ^a^	−18	−26
							193.1 → 135.9 ^b^	−18	−16
BPAF	5	335.2 → 196.7 ^b^	−4	−36	BPA-d_16_	4.8	241.3 → 141.7	−24	−26
		335.2 → 264.9 ^a^	−4	−22					
BPB	5.2	241.2 → 211.8 ^b^	−10	−16	EthPB-d_5_	2	170.2 → 91.3 ^a^	−24	−16
		241.2 → 225.9 ^a^	−10	−20			170.2 → 137.6 ^b^	−24	−14
BPP	6.1	345.2 → 315.1 ^b^	−18	−40					
		345.2 → 330.1 ^a^	−18	−24					
Voltage of capilar		3 kV			Nebulizer gas pressure		7.0 bar		
Source temperature		150 °C			Cone/desolvation gas		N_2_ (≥99.995%)		
Desolvation temperature		600 °C			Collision gas		Ar (99.999%)		
Cone gas flow		150 L h^−1^			Dwell time		25 ms		
Desolvation gas flow		500 L h^−1^			Inter-scan delay		3 ms		
Collision gas flow		0.15 mL min^−1^							

CV: Cone voltage (V); CE: Collision energy (eV). t_R_: retention time. ^a^ SRM transition used for quantification. ^b^ SRM transition used for confirmation.

**Table 2:** Validation and quality parameters of the method.

	b	Linearity		LOD	LOQ	Recovery Assay		
	g ng^−1^	R^2^	%P_lof_	ng g^−1^	ng g^−1^	Added (ng g^−1^)	%Rec	%RSD
MetPB	0.8556	0.9535	15.2	0.1	0.4	1	95	10
						100	102	5.1
						250	96	6.3
EthPB	0.9615	0.9273	22.1	0.1	0.4	1	106	8.2
						100	98	7.1
						250	103	4.8
PropPB	0.1064	0.9742	15.3	0.3	0.9	1	94	12
						100	98	6.2
						250	96	4.1
ButPB	0.2612	0.982	35.4	0.2	0.7	1	96	7.2
						100	103	3.2
						250	98	4.6
BPS	0.0626	0.9665	22.1	0.3	1	1	91	11.1
						100	92	7.4
						250	103	9.4
BPE	0.1108	0.9945	60.2	0.3	1	1	93	8.2
						100	105	2.3
						250	97	6
BPF	0.1996	0.9915	48.1	0.1	0.5	1	103	8.6
						100	104	7.2
						250	94	5.6
BPAF	0.314	0.9851	20.3	0.1	0.4	1	95	8.5
						100	103	4.1
						250	96	10.3
BPA	0.0446	0.9755	12.1	0.3	0.9	1	104	7.2
						100	104	4.5
						250	94	10.7
BPB	0.0989	0.9848	20.3	0.3	0.9	1	104	6.2
						100	97	7
						250	93	5.5
BPP	0.0019	0.9723	16.2	1	4	5	104	8.6
						100	92	5.2
						250	94	4.2

b: Slope of the calibration curve; R^2^: R-squared correlation coefficient; %P: p-value of the lack-of-fit test; LOD: Limit of detection; LOQ: Limit of quantification; %Rec: Recovery; %RSD: Relative standard deviation in percentage.

**Table 3:** Frequencies (%) and mean (ng g^−1^) of parabens and bisphenols in food samples.

	Parabens					Bisphenols							
	MetPB	EthPB	ButPB	PropPB	∑PBs	BPS	BPE	BPF	BPAF	BPA	BPB	BPP	∑BPs
Unprocessed or Minimally Processed Foods (*n* = 32)													
Frecuency (%)	70	23.3	3.3	10	73.3	46.88	6.3	0	0	21.88	0	0	63
Mean (ng g^−1^)	106.90 (95.37)	29.54 (40.06)	2.5 (1.0)	34.95 (48.0)	80.7 (81.56)	17.27 (13.7)	<LOQ	0	0	6 (3.8)	0	0	18.35 (15.47)
Processed Foods (*n* = 21)													
Frecuency (%)	42.86	23.81	9.52	4.76	52	38.1	0	0	0	38.1	0	0	67
Mean (ng g^−1^)	142.05 (120.2)	60.28 (56.2)	55.35 (42.2)	65.5 (26.2)	130.7 (87.3)	39.49 (67.0)	0	0	0	86.3 (158.6)	0	0	35 (119.5)
Ultra-Processed Foods (*n* = 47)													
Frecuency (%)	38.3	6.38	10.64	12.77	49	6.38	4.26	0	0	27.66	0	0	36
Mean (ng g^−1^)	41.75 (47.18)	28.6 (12.0)	39.87 (60.07)	3.15 (2.0)	40.31 (48.46)	47.48 (73.29)	<LOQ	0	0	35.3 (42.4)	0	0	38.34 (48.14)
All (*n* = 100)													
Frecuency (%)	49	15	8.1	10.2	57.14	26.5	4.1	0	0	28.6	0	0	52
Mean (ng g^−1^)	84.6 (88.5)	42.19 (44.71)	39.07 (50.83)	21.1 (31.5)	73.86 (76.76)	28.99 (46.29)	<LOQ	0	0	43.28 (91.45)	0	0	30.4 (41.9)

∑PBs, ∑Parabens; ∑BPs, ∑Bisphenols; LOQ. limit of quantification. (_). Standard deviation.

**Table 4:** Concentrations (ng g^−1^ fresh weight) of parabens and bisphenol analogues in unprocessed or minimally processed foods.

Sample	Packaging	Parabens					Bisphenols							
		MetPB	EthPB	ButPB	PropPB	∑PBs	BPS	BPE	BPF	BPAF	BPA	BPB	BPP	∑BPs
Chicken	Plastic and porex tray	ND	ND	ND	D		ND	ND	ND	ND	2.1 (1.0)	ND	ND	2.1 (1.0)
Eggs	Plastic and paperboard	229.9 (29.2)	ND	ND	ND	229.9 (29.2)	ND	ND	ND	ND	ND	ND	ND	
Whole milk	Plastic	ND	ND	ND	ND		ND	ND	ND	ND	ND	ND	ND	
Whole milk	Carton	ND	ND	ND	ND		ND	ND	ND	ND	ND	ND	ND	
Whole milk	Carton	ND	ND	ND	ND		ND	D	ND	ND	ND	ND	ND	
Whole milk	Carton	ND	ND	ND	ND		ND	D	ND	ND	ND	ND	ND	
Frozen hake	Plastic and paperboard	ND	ND	ND	ND		ND	ND	ND	ND	D	ND	ND	
Lentils	Plastic	ND	ND	ND	ND		ND	ND	ND	ND	D	ND	ND	
Grape	Plastic	D	ND	ND	ND		D	ND	ND	ND	ND	ND	ND	
Blueberries	Plastic	ND	ND	ND	ND		ND	ND	ND	ND	ND	ND	ND	
Pineapple	Plastic	D	5.8 (4.2)	2.5 (1.0)	68.9 (4.0)	77.2 (37.4)	44.3 (2.8)	ND	ND	ND	11.3 (4.6)	ND	ND	55.6 (23.3)
Raspberry	Plastic	ND	ND	ND	ND		ND	ND	ND	ND	ND	ND	ND	
Melon	Plastic	125.9	ND	ND	ND	125.9	4.22 (2.13)	ND	ND	ND	7.86 (4.21)	ND	ND	12.08 (2.57)
Apple	Not packed	7.4 (3.2)	ND	ND	ND	7.4 (3.2)	12.7 (1.6)	ND	ND	ND	6.0 (9.1)	ND	ND	18.7 (4.7)
Apple	Plastic	12.1 (9.2)	ND	ND	ND	12.1 (9.2)	8.5 (3.3)	ND	ND	ND	2.9 (2.4)	ND	ND	11.4 (3.96)
Pear	Not packed	101.6 (84.7)	ND	ND	ND	101.6 (84.7)	ND	ND	ND	ND	ND	ND	ND	
Frozen red fruit mix	Plastic	D	100.2 (8.2)	ND	ND	100.2 (8.2)	ND	ND	ND	ND	ND	ND	ND	
Frozen mango	Plastic	39.5	ND	ND	ND	39.5	D	ND	ND	ND	ND	ND	ND	
Frozen chopped garlic	Plastic	D	13.6 (1.3)	ND	ND	13.6 (1.3)	1.36 (0.77)	ND	ND	ND	ND	ND	ND	1.36 (0.77)
Frozen chopped onion	Plastic	231.9 (18.9)	ND	ND	ND	231.9 (18.9)	ND	ND	ND	ND	ND	ND	ND	
Frozen chopped parsley	Plastic	D	ND	ND	ND		33.3 (13.9)	ND	ND	ND	ND	ND	ND	33.3 (13.9)
Frozen spinach	Plastic	D	ND	ND	ND		D	ND	ND	ND	ND	ND	ND	
Tomato	Not packed	D	ND	ND	ND		4.7 (2.2)	ND	ND	ND	ND	ND	ND	4.7 (2.2)
Tomato	Plastic	D	ND	ND	ND		25.9 (10.7)	ND	ND	ND	ND	ND	ND	25.9 (10.7)
Striped carrot	Plastic	D	D	ND	ND		11.5 (5.3)	ND	ND	ND	ND	ND	ND	11.5 (5.3)
Carrod	Plastic	D	6 (3.6)	ND	ND	6 (3.6)	D	ND	ND	ND	ND	ND	ND	
Lettuce	Plastic	D	D	ND	ND		ND	ND	ND	ND	ND	ND	ND	
Pumpkin	Plastic	D	ND	ND	ND		ND	ND	ND	ND	ND	ND	ND	
Mushrooms	Plastic	D	22.1 (10.4)	ND	1.0 (0.1)	23.1 (14.9)	16.0 (6.9)	ND	ND	ND	ND	ND	ND	16.0 (6.9)
Green pepper	Not packed	D	ND	ND	ND		27.5 (6.3)	ND	ND	ND	ND	ND	ND	27.5 (6.3)
Mean		106.90 (95.37)	29.5 (40.6)	2.5 (1.0)	34.95 (48.0)	73.17 (79.9)	17.27 (13.7)				6 (3.8)			18.35 (15.47)

∑PBs, ∑Parabens; ∑BPs, ∑Bisphenols; ND. not detected (<LOD); D. detected (>LOD and <LOQ). (_). Standard deviation.

**Table 5:** Concentrations (ng g^−1^ fresh weight) of parabens and bisphenol analogues in processed foods.

Sample	Packaging	Parabens					Bisphenols							
		MetPB	EthPB	ButPB	PropPB	∑PBs	BPS	BPE	BPF	BPAF	BPA	BPB	BPP	∑BPs
Cooked ham	Plastic	ND	ND	ND	ND		ND	ND	ND	ND	6.6 (3.4)	ND	ND	6.6 (3.4)
Spicy Sausage	Plastic	ND	ND	ND	ND		ND	ND	ND	ND	ND	ND	ND	
Spicy Sausage	Plastic	D	ND	ND	ND		ND	ND	ND	ND	ND	ND	ND	
Chicken burguer	Plastic	191.1(118.3)	ND	ND	ND	191.1 (118.3)	ND	ND	ND	ND	ND	ND	ND	
Chicken burguer	Plastic	281.7 (88.9)	ND	ND	ND	281.7 (88.9)	ND	ND	ND	ND	ND	ND	ND	
Sausage (Chorizo)	Plastic	ND	ND	ND	ND		ND	ND	ND	ND	ND	ND	ND	
Serrano ham	Plastic	ND	ND	ND	ND		39.3 (21.3)	ND	ND	ND	17.3 (14.9)	ND	ND	56.6 (15.56)
Plain yogurt (sweetened)	Plastic	ND	ND	ND	ND		ND	ND	ND	ND	29.88 (18.6)	ND	ND	29.88 (18.6)
Plain yogurt (sweetened)	Plastic	ND	ND	ND	ND		ND	ND	ND	ND	12.3 (6.0)	ND	ND	12.3 (6.0)
Guacamole	Plastic	D	ND	ND	ND		D	ND	ND	ND	ND	ND	ND	
Olives	Plastic	5.2 (1.7)	29.2 (14.5)	ND	ND	34.4 (16.97)	8.5 (5.4)	ND	ND	ND	ND	ND	ND	8.5 (5.4)
Olives	Plastic	D	13.6 (5.5)	85.2 (39.5)	65.5 (26.2)	164.3 (36.99)	30.2 (7.7)	ND	ND	ND	ND	ND	ND	30.2 (7.7)
Anchovy stuffed olives	Can	ND	86.9 (17.5)	ND	ND	86.9 (17.5)	ND	ND	ND	ND	ND	ND	ND	
Semi-cured cheese	Plastic	ND	24.8 (7.2)	ND	ND	24.8 (7.2)	ND	ND	ND	ND	D	ND	ND	
Semi-cured cheese (slice)	Plastic	D	ND	ND	ND		5.6 (1.9)	ND	ND	ND	ND	ND	ND	5.6 (1.9)
Pasta	Plastic	ND	ND	ND	ND		ND	ND	ND	ND	ND	ND	ND	
Rice (for microwave)	Plastic and paperboard	90.2 (28.6)	ND	ND	ND	90.2 (28.6)	3.3 (1.4)	ND	ND	ND	ND	ND	ND	3.3 (1.4)
Canned tuna in oil	Can	ND	ND	ND	ND		ND	ND	ND	ND	409.0 (31.0)	ND	ND	409.0 (31.0)
Canned tuna in oil	Can	D	146.9 (8.5)	25.5 (14.9)	ND	172.4 (85.8)	187.8 (14.2)	ND	ND	ND	D	ND	ND	187.8 (14.2)
Canned sweet corn	Can	ND	ND	ND	ND		ND	ND	ND	ND	42.7 (6.2)	ND	ND	42.7 (6.2)
Cake	Not packed	ND	ND	ND	ND		1.7 (0.7)	ND	ND	ND	ND	ND	ND	1.7 (0.7)
Mean		142.05 (120.2)	60.28 (56.2)	55.35 (42.2)	65.5 (26.2)	130.7 (87.3)	39.49 (67.0)				86.3 (158.6)			35 (119.5)

∑PBs, ∑Parabens; ∑BPs, ∑Bisphenols; ND. not detected (<LOD); D. detected (>LOD and <LOQ). (_). Standard deviation.

**Table 6:** Concentrations (ng g^−1^ dw) of parabens and bisphenol analogues in ultra-processed foods.

Sample	Package Type	Parabens					Bisphenols							
		MetPB	EthPB	ButPB	PropPB	∑PBs	BPS	BPE	BPF	BPAF	BPA	BPB	BPP	∑BPs
Sausage (Hot dogs)	Plastic	6.8 (7.2)	ND	ND	ND	6.8 (7.2)	ND	ND	ND	ND	ND	ND	ND	
Turkey cold cut	Plastic	D	ND	2.05 (0.06)	ND	2.05 (0.06)	ND	ND	ND	ND	ND	ND	ND	
Sausage (Turkey cold)	Plastic	D	ND	ND	ND		ND	ND	ND	ND	ND	ND	ND	
Mortadella (Bologna)	Plastic	D	ND	16.5 (11.2)	ND	16.5 (11.2)	5.43 (3.34)	ND	ND	ND	ND	ND	ND	5.43 (3.34)
Chocolate milkshake	Carton	ND	ND	ND	ND		ND	ND	ND	ND	ND	ND	ND	
Chocolate milkshake	Carton	D	ND	ND	ND		ND	D	ND	ND	ND	ND	ND	
Chocolate milkshake	Carton	D	ND	ND	ND		ND	ND	ND	ND	ND	ND	ND	
Chocolate milkshake	Carton	ND	ND	ND	ND		ND	D	ND	ND	ND	ND	ND	
Semi-fermented milk	Plastic and foil	6.1 (3.67)	ND	ND	ND	6.1 (3.67)	ND	ND	ND	ND	ND	ND	ND	
Semi-fermented milk	Plastic and foil	41.08 (12.55)	ND	ND	ND	41.08 (12.55)	ND	ND	ND	ND	ND	ND	ND	
Semi-fermented milk	Plastic and foil	88.38 (34.9)	ND	ND	ND	88.38 (34.9)	ND	ND	ND	ND	ND	ND	ND	
Flavoured Yogurt	Plastic	26.6 (6.1)	ND	ND	ND	26.6 (6.1)	ND	ND	ND	ND	60.85 (17.2)	ND	ND	60.85 (17.2)
Flavoured Liquid Yogurt	Plastic	145.66 (8.86)	ND	ND	ND	145.66 (8.86)	ND	ND	ND	ND	115.4 (65.96)	ND	ND	115.4 (65.96)
Flavoured Liquid Yogurt	Plastic	42.49 (20.46)	ND	ND	ND	42.49 (20.46)	ND	ND	ND	ND	ND	ND	ND	
Spread cheese	Foil and paperboard	ND	ND	ND	ND		ND	ND	ND	ND	ND	ND	ND	
Melted cheese	Plastic	ND	ND	ND	ND		4.9 (0.9)	ND	ND	ND	D	ND	ND	4.9 (0.9)
Melted cheese	Plastic	ND	ND	ND	ND		ND	ND	ND	ND	2 (0.3)	ND	ND	2 (0.3)
Breadsticks for cheese	Plastic	ND	ND	ND	ND		ND	ND	ND	ND	ND	ND	ND	
Tuna dumplings	Can	ND	ND	ND	ND		ND	ND	ND	ND	ND	ND	ND	
Battered hake sticks	Plastic	ND	ND	ND	ND		ND	ND	ND	ND	ND	ND	ND	
Pizza (cooked ham and cheese)	Plastic	ND	ND	ND	ND		ND	ND	ND	ND	4.3 (1.8)	ND	ND	4.3 (1.8)
Pizza (4 cheese)	Plastic	ND	ND	ND	ND		ND	ND	ND	ND	ND	ND	ND	
Pizza (bolognese)	Plastic	ND	ND	ND	ND		ND	ND	ND	ND	ND	ND	ND	
Ketchup	Plastic	ND	ND	ND	D		ND	ND	ND	ND	ND	ND	ND	
Ketchup	Plastic	ND	ND	ND	D		ND	ND	ND	ND	D	ND	ND	
Tomato sauce	Carton	ND	ND	ND	ND		ND	ND	ND	ND	ND	ND	ND	
Corn snacks	Plastic	ND	ND	ND	1.4 (0.3)	1.4 (0.3)	ND	ND	ND	ND	ND	ND	ND	
Corn snacks	Plastic	ND	ND	ND	ND		ND	ND	ND	ND	ND	ND	ND	
Nachos	Plastic	ND	ND	ND	ND		ND	ND	ND	ND	42.1 (4.2)	ND	ND	42.1 (4.2)
Chips	Plastic	ND	ND	ND	ND		ND	ND	ND	ND	ND	ND	ND	
Chips	Plastic	ND	ND	ND	ND		ND	ND	ND	ND	ND	ND	ND	
Chips	Plastic	D	ND	ND	ND		132.1 (21.2)	ND	ND	ND	ND	ND	ND	132.1 (21.2)
Chips (Sour Cream & Onion)	Plastic, foil and paperboard	7.7 (0.7)	ND	ND	ND	7.7 (0.7)	ND	ND	ND	ND	8.8 (10.7)	ND	ND	8.8 (10.7)
Gummy candy	Plastic	ND	ND	ND	ND		ND	ND	ND	ND	ND	ND	ND	
Gummy candy	Plastic	ND	ND	ND	ND		ND	ND	ND	ND	ND	ND	ND	
Chocolate doughnuts	Plastic	ND	D	ND	ND		ND	ND	ND	ND	82 (10.2)	ND	ND	82 (10.2)
Milk bread	Plastic	ND	ND	ND	ND		ND	ND	ND	ND	ND	ND	ND	
Croissants	Plastic	ND	ND	ND	ND		ND	ND	ND	ND	ND	ND	ND	
Croissants	Plastic	D	ND	4.3 (2.6)	1.5 (0.8)	5.8 (1.98)	ND	ND	ND	ND	ND	ND	ND	
Chocolate puff pastry	Plastic	ND	ND	ND	ND		ND	ND	ND	ND	1	ND	ND	1
Cacao-filled roll	Plastic	ND	ND	ND	ND		ND	ND	ND	ND	D	ND	ND	
Muffins	Plastic	ND	ND	ND	ND		ND	ND	ND	ND	ND	ND	ND	
Muffins	Plastic	D	ND	ND	4.2 (0.8)	4.2 (0.8)	ND	ND	ND	ND	ND	ND	ND	
Burger bun	Plastic	10.9 (9.6)	20.1 (7.7)	ND	ND	31.0 (6.5)	ND	ND	ND	ND	1.2 (1.6)	ND	ND	1.2 (1.6)
Sandwich bread	Plastic	ND	ND	ND	ND		ND	ND	ND	ND	ND	ND	ND	
Milk bread with chocolate chips	Plastic	ND	ND	145.3 (10.2)	ND	145.3 (10.2)	ND	ND	ND	ND	D	ND	ND	
Puffed rice cake with chocolate	Plastic	D	37.1 (5.5)	31.2 (8.3)	5.5 (3.0)	73.8 (16.8)	ND	ND	ND	ND	ND	ND	ND	
Mean		41.75 (47.18)	28.6 (12.0)	39.87 (60.07)	3.15 (2.0)	40.31 (48.46)	47.48 (73.29)				35.3 (42.4)			38.34 (48.14)

∑PBs, ∑Parabens; ∑BPs, ∑Bisphenols; ND. not detected (<LOD); D. detected (>LOD and <LOQ). (_). Standard deviation.

## Data Availability

The data presented in this study are available in the article or supplementary material.

## Supplementary Materials

The following are available online at https://www.mdpi.com/2304-8158/10/1/92/s1, Figure S1: Chromatogram of the blank. Figure S2: Chromatogram from one of the samples (canned tuna in oil), which contains remarkable levels of BPA, BPS, MetPB, EthPB and ButPB. Table S1: Concentrations (ng g^−1^ or ng mL^−1^ dw) of parabens found in food from countries of the European Union. Table S2: Concentrations (ng g^−1^ or ng mL^−1^ dw) of bisphenols found in food samples from European Union countries. Table S3: Estimated dietary intake of parabens in Spanish children aged 6–9 years. Table S4: Estimated dietary intake of bisphenols in Spanish children aged 6–9 years.

## Abbreviations

BPA	Bisphenol A
AESAN	Spanish Agency for Food Safety and Nutrition
BPA-d_16_	Deuterium labelled bisphenol A
BPAF	Bisphenol AF
BPAP	Bisphenol AP
BPB	Bisphenol B
BPE	Bisphenol E
BPF	Bisphenol F
BPM	Bisphenol M
BPP	Bisphenol P
BPS	Bisphenol S
BPZ	Bisphenol Z
ButPB	Butyl 4-hydroxybenzoate
bw	Body weight
C18	Octadecyl-functionalized silica
ECHA	European Chemical Agency
EFSA	European Food Safety Authority
ESI	Electrospray ionization source
EthPB	Ethyl 4-hydroxybenzoate
EthPB-d_5_	Deuterium labelled ethylparaben
LC-MS	Liquid chromatography-mass spectrometry
LLE	Liquid-liquid extraction
LOD	Limit of detection
LOQ	Limit of quantification
MeOH	Methanol
MetPB	Methyl 4-hydroxybenzoate
MgSO_4_	Magnesium sulphate
MPLs	Maximum permitted levels
MRM	Multiple-reaction-monitoring
NaCl	Sodium chloride
PropPB	Propyl 4-hydroxybenzoate
PSA	Primary Secondary Amine
QuEChERs	Quick, Easy, Cheap, Effective, Rugged, and Safe
SCCS	Scientific Committee on Consumer Safety
SPE	Solid phase extraction
TDI	Tolerable dietary intake
UHPLC-MS/MS	Ultra-high performance liquid chromatography-tandem mass spectrometry
