# Supplementary material for: Presence of Parabens and Bisphenols in Food Commonly Consumed in Spain
Source: Foods. 2021 Jan 5;10(1):92. doi: 10.3390/foods10010092 (PMC7824906; doi:10.3390/foods10010092)
Supplement: Supplementary file 1 [file foods-10-00092-s001.pdf]

foods

Supplementary materials

PRESENCE OF PARABENS AND BISPHENOLS IN FOOD COMMONLY CONSUMED IN SPAIN

Yolanda Gálvez-Ontiveros[1,2], Inmaculada Moscoso-Ruiz[3], Lourdes Rodrigo[4], Margarita Aguilera[2,5], Ana Rivas[1,2,*] and Alberto Zafra-Gómez[2,3]

[1] Department of Nutrition and Food Science, University of Granada, Campus of Cartuja, 18071 Granada, Spain.
[2] Instituto de Investigación Biosanitaria. Ibs-Granada.
[3] Department of Analytical Chemistry, University of Granada, Campus of Fuentenueva, 18071 Granada, Spain.
[4] Department of Legal Medicine and Toxicology, University of Granada, 18071 Granada, Spain.
[5] Department of Microbiology, Faculty of Pharmacy, University of Granada, Campus of Cartuja, 18071 Granada, Spain.
* Corresponding author: amrivas@ugr.es; Tel: +34 958 242841; Fax: +34 958 249577

Number of pages: 20; Number of figures: 2; Number of tables: 4

The supplementary materials contain supplementary figures and tables for Material and Methods and Discussion.

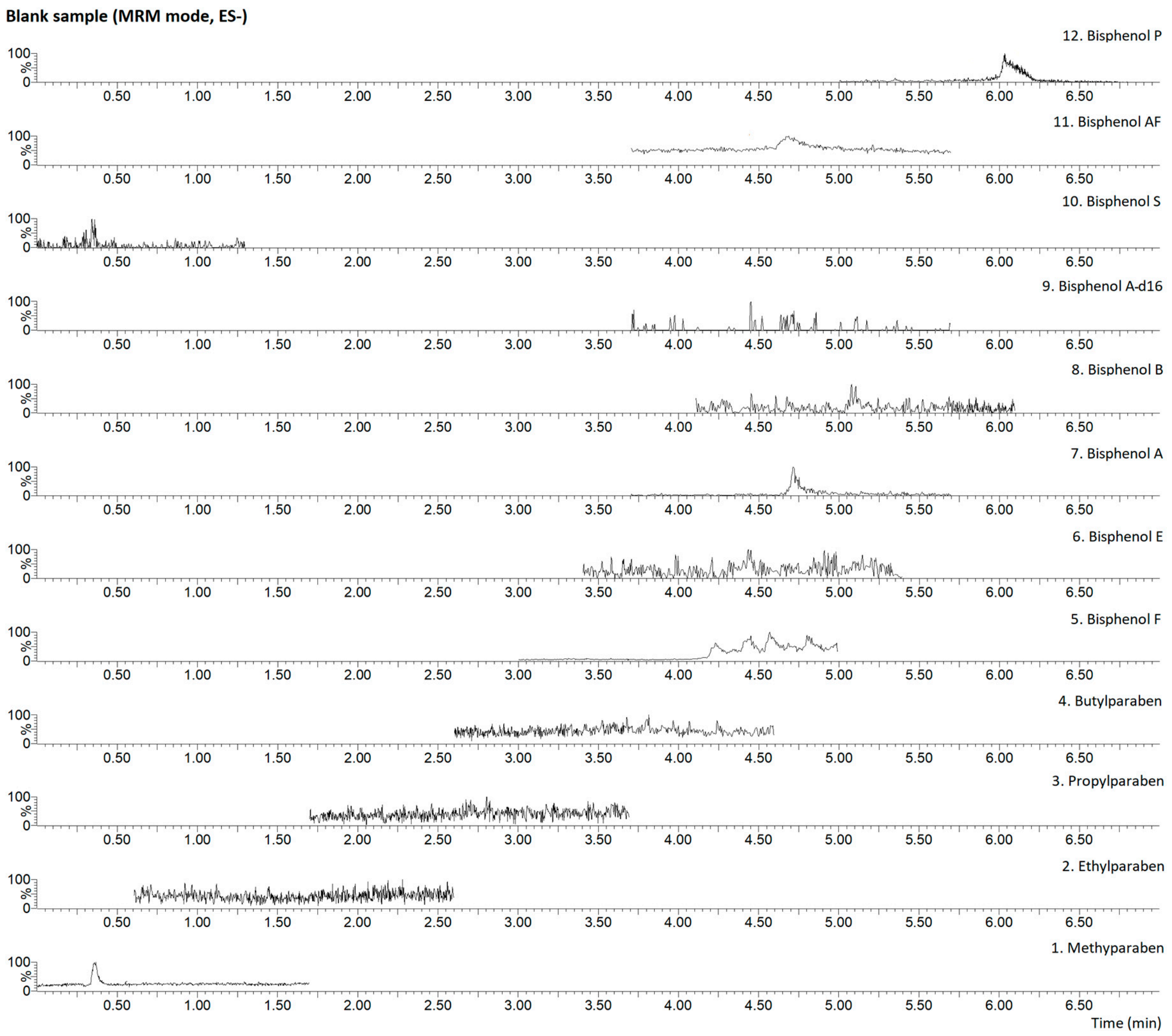

Figure S1. Chromatogram of the blank

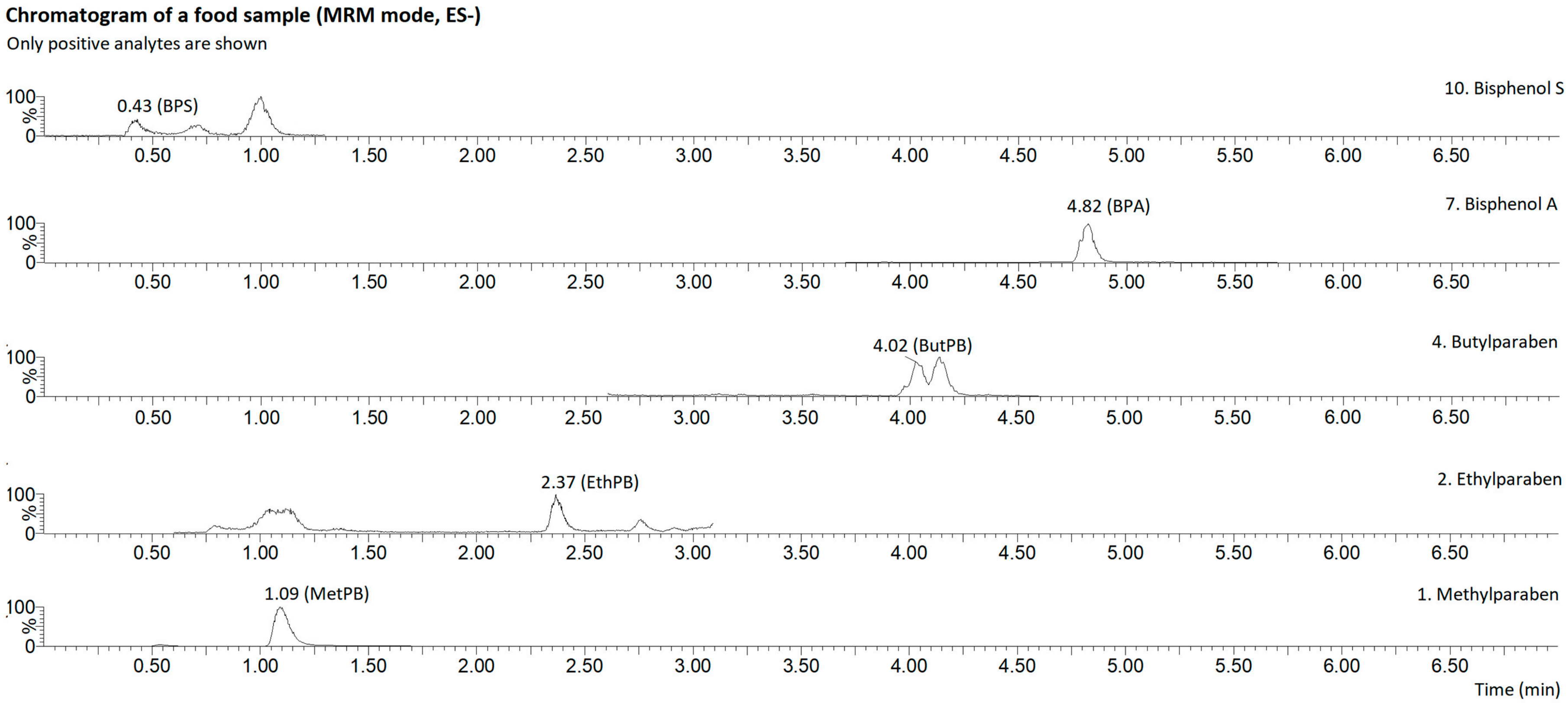

Figure S2. Chromatogram from one of the samples (canned tuna in oil), which contains remarkable levels of BPA, BPS, MetPB, EthPB and ButPB.

Table S1. Concentrations (ng g^{-1} or ng mL^{-1} dw) of parabens found in food from countries of the European Union.

	Samples	*Location*	*EDCs*	*Separation and detection technique*	*LOD*	*LOQ*	*Minimum concentration*	*Mean concentration*	*Maximum concentration*	*Reference*
Fresh food										
Fresh fish	Bivalves (mussels, clams and oysters) and fish (mullet and flounder)	Europe (Portugal, Spain, Italy, the Netherlands and Norway)	Methylparaben (MetPB)	UHPLC-QqQ-MS	-	-	<LOQ	8.64 ± 5.9	16.4 ± 1.7	Álvarez-Muñoz et al., 2015 [38]
			Ethylparaben (EthPB)		-	-	<LOQ	0.23 ± 0.15	0.4 ± 0.03	
			Propylparaben (PropPB)		-	-	<LOQ	1.31 ± 0.91	2.8 ± 0.1	
			Benzylparaben (BzPB)		-	-	<LOQ	–	–	
	Fish (Mackerel and Plaice) and Bivalves (mussels)	Europe (Portugal, Spain, Italy, Greece, The Netherlands, United Kingdom (Scotland), Denmark, Norway, Belgium, France and Ireland)	MetPB	UHPLC-QqQ-MS	0.005-0.04	0.01-0.12	<LOD	3.35 ± 2.21	8.86 ± 0.1	Álvarez-Muñoz et al., 2018 [39]
	Fish (Anchovies, Sardines, Hake, Cod, Salmon, Trout, Sea Bass and Bream) and seafood (Squid, Cuttlefish, Octopus, Shrimp, Mussels, Clams and Oysters)	Europe (Portugal, Spain, Italy, Greece and Norway)	MetPB	GC-MS/MS	0.0041	-	<LOD	0.1 ± 0.06	0.16 ± 0.01	Azzouz et al., 2019 [40]
			EthPB		0.0042	-	<LOD	0.07 ± 0.07	0.21 ± 0.01	
			Isopropylparaben		0.0028	-	<LOD	0.23 ± 0.16	0.44 ± 0.03	
	54 species of fish and 10 species of bivalves	Italy	MetPB	HPLC- benchtop Q Exactive Orbitrap high-resolution MS	0.65	2.15	0.8	6.4	32	Chiesa et al., 2018 [41]
			EthPB		1.2	3.9	<LOD	–	–	
			PropPB		0.8	2.7	<LOD	–	–	

			Butylparaben (ButPB)		0.9	2.9	<LOD	–	–	
			BzPB		0.8	2.75	<LOD	–	–	
	Bivalve molluscs (Mussels and clams)	Spain	MetPB	LC-MS/MS	0.43	1.43	<LOQ	2.94 ± 2.29	7.00 ± 2.00	Villaverde-de-Sáa et al., 2016 [42]
			EthPB		0.07	0.23	<LOQ	0.31 ± 0.04	0.37 ± 0.08	
			PropPB		0.16	0.55	<LOQ	–	0.56 ± 0.01	
			ButPB		0.04	0.12	<LOD	–	–	
	Fish (Graells barbel, largemouth bass, carp, trout, catfish, eel, percasol, goby, gypsy barbel, alburno, Guadiana boga)	Spain	MetPB	UHPLC-MS/MS	0.005-0.04	0.01-0.12	<LOD	11.43 ± 22.00	84.69 ± 6.58	Jakimska et al., 2013 [43]
			EthPB		0.004-0.05	0.01-0.14	<LOD	0.8 ± 0.03	0.82	
			PropPB		0.002-0.01	0.005-0.02	<LOD	2.47 ± 3.06	7.43 ± 0.69	
			BzPB		0.003-0.02	0.01-0.06	<LOD	0.39 ± 0.07	0.54	
	Barbus graellsii, Micropterus salmoides, Cyprinus carpio, Salmo trutta, Silurus glanis, Anguilla anguilla, Lepomis gibbosus, Gobio gobio, Luciobarbus sclateri, Aburnus alburnus, and Pseudochondrostoma willkommii.	Spain	MetPB	LC-MS/MS	0.0005-5	0.0015-15	<LOD	3.68	84.69	Pico et al., 2019 [44]
			EthPB				<LOD	0.06	0.82	
			PropPB				<LOD	0.32	7.43	
			BzPB				<LOD	0.07	0.54	
Fresh vegetables	Tubers (carrot, turnip and potato)	Spain	MetPB	LC-MS/MS	0.15	0.5	<LOD	–	–	Abril et al., 2018 [45]
			EthPB		0.03	0.1	<LOD	–	–	
			PropPB		0.03	0.1	<LOD	–	–	

			BzPB		0.03	0.1	<LOD	–	–	
Cooked packaged food										
Cooked fish	Fish (Cod, Mackerel, Sole, Dorada, Bream and Salmon), bivalves (Mussels) and crustaceans (Brown Crab)	Europe (Portugal, Spain, Italy, Greece, The Netherlands, United Kingdom (Scotland), Denmark, Norway, Belgium, France and Ireland)	MetPB	UHPLC-QqQ-MS	0.005-0.04	0.01-0.12	<LOD	16.14 ± 9.86	25.53 ± 1.1	Álvarez-Muñoz et al., 2018 [39]
	Cooked prawns and crabs	Europe (Portugal, Spain, Italy)	MetPB	GC-MS/MS	0.0041	-	<LOD	–	0.03 ± 0.002	Azzouz et al., 2019 [40]
			EthPB		0.0041	-	<LOD	–	0.04 ± 0.002	
			Isopropylparaben		0.0028	-	<LOD	–	0.65 ± 0,03	
Canned food										
Canned fish	Fish (Tuna and Mackerel)	Europe (Portugal, Spain, Italy, Greece, The Netherlands, United Kingdom (Scotland), Denmark, Norway, Belgium, France and Ireland)	MetPB	UHPLC-QqQ-MS/MS	0.005-0.04	0.01-0.12	<LOD	–	–	Álvarez-Muñoz et al., 2018 [39]

LOD. Limit of detection; LOQ. limit of quantification; ***UHPLC-QqQ-MS. Ultra high performance liquid chromatography–triple quadrupole mass spectrometry; GC-MS/MS. Gas chromatography–tandem mass spectrometry; HPLC- benchtop Q Exactive Orbitrap high-resolution MS. High-performance liquid chromatograph, coupled with a benchtop Q Exactive Orbitrap high-resolution MS; LC-MS/MS. Liquid chromatography-tandem mass spectrometry; UHPLC-MS/MS; Ultra high performance liquid chromatography-tandem mass.***

Table S2. Concentrations (ng g^{-1} or ng mL^{-1} dw) of bisphenols found in food samples from European Union countries.

	Samples	*Location*	*EDCs*	*Separation and detection technique*	*LOD*	*LOQ*	*Minimum concentration*	*Mean concentration*	*Maximum concentration*	*Reference*
Fresh food										
Fresh fish	Bivalves (mussels, clams and oysters) and fish (mullet and flounder)	Europe (Portugal, Spain, Italy, the Netherlands and Norway)	Bisphenol A (BPA)	UHPLC-QqQ-MS	-	-	<LOQ	8.44 ± 2.72	15 ± 1.8	Álvarez-Muñoz et al., 2015 [38]
	Fish (Mackerel and Plaice) and Bivalves (mussels)	Europe (Portugal, Spain, Italy, Greece, The Netherlands, United Kingdom (Scotland), Denmark, Norway, Belgium, France and Ireland)	BPA	UHPLC-QqQ-MS	0.008-0.06	0.03-0.20	<LOD	12.55 ± 6.71	25.02 ± 0.9	Álvarez-Muñoz et al., 2018 [39]
	Fish (Anchovies, Sardines, Hake, Cod, Salmon, Trout, Sea Bass and Bream) and seafood (Squid, Cuttlefish, Octopus, Shrimp, Mussels, Clams and Oysters)	Europe (Portugal, Spain, Italy, Greece and Norway)	BPA	GC-MS/MS	0.0006	-	<LOD	0.05 ± 0.04	0.13 ± 0.01	Azzouz et al., 2019 [40]
	50 species of fish (muscle and liver)	Portugal	BPA	GC-MS/MS	0.9-1.8	1.3-3.6	1 ± 3.9	10.03 ± 8.22	25.3 ± 63.50	Barboza et al., 2020 [79]
			Bisphenol AP (BPAP)		2.4-5.5	3.0-7.7	<LOD	–	–	
			Bisphenol AF (BPAF)		1.7-5.8	2.6-7.3	<LOD	–	–	
			Bisphenol B (BPB)		0.9-2.2	1.3-3.6	0.7 ± 5.1	7.3 ± 8.91	19.9 ± 47.20	
			Bisphenol E (BPE)		1.5-5.1	2.2-7.7	0.022 ± 0.2	1.53 ± 1.82	4.1 ± 22.00	
			Bisphenol F (BPF)		1.9-5.5	2.6-7.3	<LOD	–	–	
			Bisphenol Z (BPZ)		1.65-5.5	2.4-7.7	<LOD	–	–	

	Red mullet (liver and muscle)	Italia	BPA	HPLC- FLD	0.1	1	35.00 ± 10.8	52.5 ± 12.3	77.6 ± 6.5	Errico et al., 2017 [67]
	Salmon and squid	Spain	BPA	GC-MS	0.33	-	<LOD	–	–	González et al., 2020 [63]
			BPB		0.33	-	<LOD	–	–	
			BPE		0.83	-	<LOD	–	–	
	Barbus graellsii, Micropterus salmoides, Cyprinus carpio, Salmo trutta, Silurus glanis, Anguilla anguilla, Lepomis gibbosus, Gobio gobio, Luciobarbus sclateri, Aburnus alburnus, and Pseudochondrostoma willkommii.	Spain	BPA	UHPLC-MS/MS	0.003-0.01	0.008-0.04	<LOD	141.5 ± 116.55	223.91 ± 11.51	Jakimska et al., 2013 [43]
Fresh meat	Chicken	Spain	BPA	GC-MS	–	0.33	–	–	1.41	González et al., 2020 [63]
			BPB		–	0.33	–	–	4.19	
			BPE		–	0.83	<LOD	–	–	
Fresh vegetables	Tubers (carrot, turnip and potato)	Spain	BPA	LC-MS/MS	1.5	5	<LOD	–	–	Abril et al., 2018 [45]
Fresh fruit	Banana	Spain	BPA	GC-MS	–	0.17	<LOD	–	–	González et al., 2020 [63]
			BPB		–	0.17	<LOD	–	–	
			BPE		–	0.17	<LOD	–	–	
Cooked packaged food										
Cooked fish	Fish (Cod, Mackerel, Sole, Dorada, Bream and Salmon), bivalves (Mussels) and crustaceans (Brown Crab)	Europe (Portugal, Spain, Italy, Greece, The Netherlands, United Kingdom (Scotland), Denmark, Norway, Belgium, France and Ireland)	BPA	UHPLC-QqQ-MS	0.008-0.06	-	<LOD	39.25 ± 20.75	54.7 ± 1.7	Álvarez-Muñoz et al., 2018 [39]
	Cooked prawns and crabs	Europe (Portugal, Spain, Italy)	BPA	GC-MS/MS	0.0006	-	<LOD	–	0.0046 ± 0.0003	Azzouz et al., 2019 [40]
Cooked and packaged foods	Pasta with tomato sauce and cheese, rice with seafood and vegetables, and chicken and vegetables.	Spain	BPA	GC–MS	7.10	-	26.00	103.83 ± 81.78	218.00	Fasano et al., 2015 [73]
Canned food										
Canned fish	Fish (mackerel) and bivalves (mussels and cockles)	Spain	BPA	LC-FD	0.8	2.9	<LOD	149.00 ± 46.00	182.00 ± 6.00	Alabi et al., 2014 [71]

			BPB		0.6	2.0	<LOD	–	40.00 ± 3.00	
			BPE		1.1	3.5	<LOD	–	–	
			BPF		0.8	2.6	<LOD	–	–	
			Bisphenol F diglycidyl-ether (BFDGE) and derivatives		0.3-1.0	1.2-3.2	<LOD	–	251.00 ± 1.00	
			Bisphenol A diglycidyl-ether (BADGE) and derivatives		0.3-0.5	0.9-1.6	<LOD	160.00 ± 208.00	662.00 ± 6.00	
	Fish (Tuna and Mackerel)	Europe (Portugal, Spain, Italy, Greece, The Netherlands, United Kingdom (Scotland), Denmark, Norway, Belgium, France and Ireland)	BPA	UHPLC-QqQ-MS	0.008-0.06	0.03-0.20	7.6 ± 1.5	32.68 ± 27.03	69.1 ± 11.82	Álvarez-Muñoz et al., 2018 [39]
	Fish (tuna in vegetable oil, tuna in olive oil, tuna in brine, tuna with isoflavones, tuna in tomato sauce, anchovies in vegetable oil, cod in vegetable oil and garlic, eels in pickled sauce, mackerel in vegetable oil, mackerel in tomato, pickled mussels, octopus with garlic, octopus stew, sardines in vegetable oil, sardines in spicy vegetable oil, sardines in tomato sauce, sardines in piripiri sauce, stuffed squid and squid stew)	Portugal	BPA	GC-MS	0.2	-	<LOD	19.07 ± 22.35	99.9	Cunha et al., 2012 [80]
			BPB		0.4	-	<LOD	–	21.7	

	Tuna in olive oil and natural tuna (tuna and supernatant are analysed)	Italy	BPA	LC-FD	1.3	4.3	<LOD	62.52 ± 44.21	187.00 ± 4.40	Fattore et al., 2015 [81]
			BPB		3.0	10.0	<LOD	78.13 ± 52.02	145.9 ± 5.30	
			BFDGE		3.7	12.3	<LOD	–	38.50 ± 4.90	
			BADGE		2.2	7.5	<LOD	53.68 ± 26.20	91.10 ± 3.90	
	Squid, tuna and mackerel	Spain	BPA	GC-MS	–	0.33	30.85	32.09 ± 1.18	33.19	González et al., 2020 [63]
			BPB		–	0.33	<LOD	–	–	
			BPE		–	0.83	<LOD	–	–	
Canned meat	Meat products (tripe and meatballs)	Spain	BPA	LC-FD	0.8	2.9	62.00 ± 2.00	72.00 ± 14.14	82.00 ± 3.00	Alabi et al., 2014 [71]
			BPB		0.6	2.0	<LOD	–	39.00 ± 1.00	
			BPE		1.1	3.5	<LOD	–	–	
			BPF		0.8	2.6	<LOD	–	–	
			BFDGE and derivatives		0.3-1.0	1.2-3.2	<LOD	277.00 ± 52.33	314.00 ± 1.00	
			BADGE and derivatives		0.3-0.5	0.9-1.6	<LOD	167.00 ± 200.48	630.00 ± 24.00	
	Pate and chicken	Spain	BPA	GC-MS	–	–	13.39	17.15 ± 5.32	20.91	González et al., 2020 [63]
			BPB		–	0.33	<LOD	–	3.86	
			BPE		–	0.83	<LOD	–	–	
Canned vegetables	Mushrooms, red pepper, green beans and asparagus	Spain	BPA	LC-FD	0.8	2.9	60.00 ± 3.00	137.75 ± 78.74	241.00 ± 2.00	Alabi et al., 2014 [71]
			BPB		0.6	2.0	<LOD	–	25.00 ± 1.00	
			BPE		1.1	3.5	<LOD	–	–	
			BPF		0.8	2.6	<LOD	–	–	

			BFDGE and derivatives		0.3-1.0	1.2-3.2	<LOD	79.33 ± 95.97	190.00 ± 13.00	
			BADGE and derivatives		0.3-0.5	0.9-1.6	<LOD	186.90 ± 258.58	959.00 ± 18.00	
	Supernatant (mushroom, mushroom, asparagus, artichokes, olives and pepper)	Spain	BPA	TD-GC-MS	0.0025	0.0084	3.83 ± 9.11	6.90 ± 3.67	13.98 ± 0.30	Cacho et al., 2012 [74]
			BPF		0.0009	0.0032	0.87 ± 0.03	2.64 ± 2.97	7.07 ± 0.02	
			BPZ		0.0017	0.0057	<LOD	–	–	
			BP (bisphenol)		0.0009	0.0031	<LOD	–	–	
	Asparagus and red pepper	Spain	BADGE	LC-MS/MS	0.15	0.5	<LOD	–	–	Gallart-Ayala et al., 2011 [26]
			BADGE·2 H_2O		0.15	0.5	157.00 ± 25.00	416.00 ± 366.28	675.00 ± 100.00	
			BADGE·H_2O		0.15	0.5	35.00 ± 7.00	44.00 ± 12.73	53.00 ± 11.00	
			BADGE·HCl·H_2O		0.15	0.5	4.70 ± 1.00	139.00 ± 190.42	274.00 ± 40.00	
			BADGE·HCl		0.15	0.5	<LOD	–	11.00 ± 1.50	
			BADGE·2 HCl		8	2.5	1.60 ± 0.10	2.20 ± 0.85	2.80 ± 0.20	
			BFDGE		0.15	0.5	<LOD	–	–	
			BFDGE·2 H2O		8	2.5	<LOD	–	–	
			BFDGE·2 HCl		8	2.5	<LOD	–	–	
	Mushrooms, Asparagus, Artichokes and Greens beans.	Spain	BPA	GC-MS	–	0.17	6.31	31.97 ± 38.20	88.66	González et al., 2020 [63]
			BPB		–	0.17	<LOD	–	–	
			BPE		–	0.17	<LOD	–	–	
Canned	Chickpeas and lentils	Spain	BPA	LC-FD	0.8	2.9	83.00 ± 1.00	99.5 ± 23.33	116.00 ± 4.00	Alabi et al., 2014 [71]

legumes			BPB		0.6	2.0	<LOD	–	–	
			BPE		1.1	3.5	<LOD	–	–	
			BPF		0.8	2.6	<LOD	–	–	
			BFDGE and derivatives		0.3-1.0	1.2-3.2	<LOD	54.33 ± 56.87	120.00 ± 10.00	
			BADGE and derivatives		0.3-0.5	0.9-1.6	<LOD	175.00 ± 194.04	616.00 ± 40.00	
	Supernatant (Peas)	Spain	BPA	TD-GC-MS	0.0025	0.0084	<LOD	–	–	Cacho et al., 2012 [74]
			BPF		0.0009	0.0032	<LOD	–	4.44 ± 0.22	
			BPZ		0.0017	0.0057	<LOD	–	–	
			BP		0.0009	0.0031	<LOD	–	0.58 ± 0.07	
	Red beans	Spain	BPA	GC-MS	–	–	–	–	26.16	González et al., 2020 [63]
			BPB		–	0.17	<LOD	–	–	
			BPE		–	0.17	<LOD	–	–	
Canned fruit	Pineapple and peach	Spain	BPA	LC-FD	0.8	2.9	<LOD	–	13.00 ± 1.00	Alabi et al., 2014 [71]
			BPB		0.6	2.0	<LOD	–	–	
			BPE		1.1	3.5	<LOD	–	–	
			BPF		0.8	2.6	<LOD	–	–	
			BFDGE and derivatives		0.3-1.0	1.2-3.2	<LOD	–	–	
			BADGE and derivatives		0.3-0.5	0.9-1.6	<LOD	–	6.10 ± 0.20	
	Supernatant (Pineapple)	Spain	BPA	TD-GC-MS	0.0025	0.0084	1.51 ± 0.11	3.43 ± 2.72	5.35 ± 0.17	Cacho et al., 2012 [74]
			BPF		0.0009	0.0032	<LOD	–	–	
			BPZ		0.0017	0.0057	0.76 ± 0.1	0.84 ± 0.11	0.92 ± 0.12	

			BP		0.0009	0.0031	<LOD	–	–	
	Pineapple	Spain	BADGE	LC-MS/MS	0.15	0.5	<LOD	–	–	Gallart-Ayala et al., 2011 [26]
			BADGE·2 H_2O		0.15	0.5	2.8 ± 0.10	2.95 ± 0.21	3.10 ± 0.60	
			BADGE·H_2O		0.15	0.5	<LOD	–	–	
			BADGE·HCl·H_2O		0.15	0.5	<LOD	–	–	
			BADGE·HCl		0.15	0.5	<LOD	–	–	
			BADGE·2 HCl		8	2.5	<LOD	–	0.90 ± 0.10	
			BFDGE		0.15	0.5	<LOD	–	–	
			BFDGE·2 H2O		8	2.5	<LOD	–	–	
			BFDGE·2 HCl		8	2.5	<LOD	–	–	
	Fruit salad and peach in syrup and nuts	Spain	BPA	GC-MS	–	0.17	4.49	8.09 ± 5.09	11.69	González et al., 2020 [63]
			BPB		–	0.17	<LOD	–	–	
			BPE		–	0.17	<LOD	–	–	
Canned cereals	Corn	Spain	BPA	LC-FD	0.8	2.9	–	–	142.00 ± 2.00	Alabi et al., 2014 [71]
			BPB		0.6	2.0	<LOD	–	–	
			BPE		1.1	3.5	<LOD	–	–	
			BPF		0.8	2.6	<LOD	–	–	
			BFDGE and derivatives		0.3-1.0	1.2-3.2	<LOD	–	–	
			BADGE and derivatives		0.3-0.5	0.9-1.6	<LOD	42.10 ± 42.60	91.00 ± 4.00	
	Supernatant (Corn)	Spain	BPA	TD-GC-MS	0.0025	0.0084	–	–	2.45 ± 0.04	Cacho et al., 2012 [74]
			BPF		0.0009	0.0032	<LOD	–	–	
			BPZ		0.0017	0.0057	<LOD	–	–	

			BP		0.0009	0.0031	<LOD	–	–	
	Corn	Spain	BADGE	LC-MS/MS	0.15	0.5	<LOD	–	–	Gallart-Ayala et al., 2011 [26]
			BADGE·2 H_2O		0.15	0.5	252.00 ± 19.00	310.50 ± 82.73	369.00 ± 18.00	
			BADGE·H_2O		0.15	0.5	37.00 ± 6.00	38.50 ± 2.12	40.00 ± 1.00	
			BADGE·HCL·H_2O		0.15	0.5	3.40 ± 0.70	3.90 ± 0.71	4.40 ± 0.80	
			BADGE·HCL		0.15	0.5	<LOD	–	–	
			BADGE·2 HCL		8	2.5	1.1 ± 0.10	1.90 ± 1.13	2.70 ± 0.30	
			BFDGE		0.15	0.5	<LOD	–	–	
			BFDGE·2 H2O		8	2.5	<LOD	–	–	
			BFDGE·2 HCl		8	2.5	<LOD	–	–	
	Corn	Spain	BPA	GC-MS	–	–	–	–	10.65	González et al., 2020 [63]
			BPB		–	0.17	<LOD	–	–	
			BPE		–	0.17	<LOD	–	–	
Canned beverages	Tonic, sports drink, cola, tea, soda and beer.	Spain	BPA	TD-GC-MS	0.0025	0.0084	<LOD	0.43 ± 0.2	0.68 ± 0.02	Cacho et al., 2012 [74]
			BPF		0.0009	0.0032	<LOD	0.14 ± 0.1	0.26 ± 0.01	
			BPZ		0.0017	0.0057	<LOD	–	0.09 ± 0.01	
			BP		0.0009	0.0031	<LOD	–	–	
	Beer	Italy	BPA	UHPLC-FD	0.15	0.5	<LOQ	0.62 ± 0.12	0.80 ± 0.20	Cirillo et al., 2019 [75]
			BPB		0.15	0.5	<LOQ	–	–	
			BPF		0.15	0.5	<LOQ	1.58 ± 0.55	2.50 ± 0.50	
			BADGE		0.15	0.5	<LOQ	–	–	
			BFDGE		0.15	0.5	<LOQ	–	–	
	Cola drink, lemon soda, orange soda, beer and tea	Spain	BADGE	LC-MS/MS	0.15	0.5	<LOD	–	–	Gallart-Ayala et al., 2011 [26]
			BADGE·2 H_2O		0.15	0.5	2.10 ± 0.10	3.36 ± 1.12	5.10 ± 0.60	

			BADGE·H $_2$O		0.15	0.5	<LOD	–	–	
			BADGE·HCL·H_2O		0.15	0.5	<LOD	–	–	
			BADGE·HCL		0.15	0.5	<LOD	–	–	
			BADGE·2HCL		8	2.5	<LOD	–	–	
			BFDGE		0.15	0.5	<LOD	–	–	
			BFDGE·2H2O		8	2.5	<LOD	–	–	
			BFDGE·2HCl		8	2.5	<LOD	–	–	
	Energy drink	Italy	BPA	UHPLC-FD	0.15	0.5	<LOQ	1.18 ± 0.83	3.3	Gallo et al., 2017 [20]
			BPB		0.15	0.5	<LOQ	-–	–	
			BPF		0.15	0.5	<LOQ	0.82 ± 0.38	1.3	
			BFDGE		0.15	0.5	<LOQ	0.55 ± 0.06	0.6	
			BADGE		0.15	0.5	<LOQ	4.70 ± 6.86	19.4	
	Beer and energy drink	Italy	BPA	High-resolution multiple stage mass spectrometry analyses	0.0002-9.3	0.0031-0.54	<LOD	23.09 ± 24.14	76.46 ± 1.20	Russo et al., 2019 [25]
			BPAF		0.0002-0.05	0.0031-0.167	<LOD	148.52 ± 54.92	283.53 ± 1.80	
			BPB		0.0002-0.05	0.0031-0.167	<LOD	44.99 ± 69.44	183.20 ± 1.50	
			BPE		0.0002-0.05	0.0031-0.167	<LOD	–	58.75 ± 2.00	
			BPF		0.0002-0.05	0.0031-0.167	<LOD	59.50 ± 34.98	139.26 ± 5.00	
			BPM		0.0002-0.05	0.0031-0.167	<LOD	283.9 ± 393.63	1358.32 ± 3.00	
			BADGE		0.15	0.5-4000	<LOD	38.83 ± 17.49	113.74 ± 3.00	

Oil	Olive oil	Spain	BPA	GC-MS	–	0.17	<LOD	–	–	González et al., 2020 [63]
			BPB		–	–	–	–	1.25	
			BPE		–	0.83	<LOD	–	–	
Packaged food										
Packaged fish and derivatives	Fish broth (tetrapack)	Spain	BPA	GC-MS	7.10	–	0.87	1.10 ± 0.32	1.32	Fasano et al., 2015 [73]
	Tuna (glass)	Spain	BPA	GC-MS	–	–	–	–	5.68	González et al., 2020 [63]
			BPB		–	0.33	<LOD	–	–	
			BPE		–	0.83	<LOD	–	–	
Packaged meat and derivatives	Meat broth (tetrapack)	Spain	BPA	GC-MS	7.10	–	0.76	0.87 ± 0.15	0.97	Fasano et al., 2015 [73]
	Pate (glass)	Spain	BPA	GC-MS	–	–	–	–	5.10	González et al., 2020 [63]
			BPB		–	0.33	<LOD	–	–	
			BPE		–	0.83	<LOD	–	–	
Packaged dairy and derivatives	sterilized milk (HDPE bottle), UHT milk (tetra brik)	Spain	BPA	GC-MS	0.15	-	0.99 ± 0.10	1.52 ± 0.76	2.64 ± 0.60	Casajuana et al., 2004 [66]
			BADGE		0.36	-	<LOD	–	–	
	Milk stored in PET, Tetra-Pak, HDPE and Tetra Brik	Italy	BPA	LC-FD	2.5	8.0	<LOD	108.67 ± 164.77	521.00 ± 0.80	Grumetto et al., 2013 [77]
			BPB		3.0	10.0	<LOD	33.00 ± 29.44	67.00 ± 4.00	
			BPF		0.3	1.0	<LOD	10.08 ± 8.45	26.20 ± 1.70	
	Skimmed milk powder	Spain and Italy	BPA	LC-MS/MS	50	600	<LOD	–	800.00	Ferrer et al., 2010 [68]
	Yogurt (plastic and glass)	Spain	BPA	GC-MS	–	0.17	<LOD	–	–	González et al., 2020 [63]
			BPB		–	0.17	<LOD	–	–	
			BPE		–	0.17	<LOD	–	–	
Packaged vegetables and derivatives	Vegetables broth (tetrapack)	Spain	BPA	GC-MS	7.1	–	0.72	0.77 ± 0.07	0.82	Fasano et al., 2015 [73]
	Mushrooms, asparagus, artichokes (glass), salad and green beans (plastic)	Spain	BPA	GC-MS	–	0.17	<LOD	–	9.56	González et al., 2020 [63]
			BPB		–	0.17	<LOD	–	–	
			BPE		–	0.17	<LOD	–	2.40	
	Red beans (glass)	Spain	BPA	GC-MS	–	–	–	–	8.78	González et al.,

Packaged legumes and derivatives			BPB		–	0.17	<LOD	–	–	
			BPE		–	0.17	<LOD	–	–	
Packaged fruit and derivatives	Fruit salad, peach in syrup (glass) and nuts (plastic)	Spain	BPA	GC-MS	–	0.17	<LOD	–	3.85	González et al., 2020 [63]
			BPB		–	0.17	<LOD	–	–	
			BPE		–	0.17	<LOD	–	12.35	
	Corn (glass), rice and quinoa (plastic)	Spain	BPA	GC-MS	–	0.17	<LOD	–	4.21	González et al., 2020 [63]
			BPB		–	0.17	<LOD	–	–	
			BPE		–	0.17	<LOD	–	–	
Packaged beverages	White wine (tetrapack)	Spain	BPA	GC-MS	0.71	–	0.77	0.89 ± 0.18	1.02	Fasano et al., 2015 [73]
	Red wine (tetrapack)	Spain	BPA	GC-MS	0.71	–	<LOD	–	–	Fasano et al., 2015 [73]
Packaged pre-cooked food	Pasta with tomato sauce and cheese, rice with seafood and vegetables, and chicken and vegetables, kept in a microwave-safe container made of polypropylene and polycarbonate plastic.	Spain	BPA	GC-MS	7.1	–	20.00	83.67 ± 55.38	185.00	Fasano et al., 2015 [73]
	Rice and quinoa (plastic)	Spain	BPA	GC-MS	–	0.17	1.04	1.98 ± 1.34	2.93	González et al., 2020 [63]
			BPB		–	0.17	<LOD	–	–	
			BPE		–	0.17	<LOD	–	–	
Packaged honey	Honey	Europe (Slovenia and Greece)	BPA	GC-MS	0.128	0.428	<LOD	21.00 ± 31.70	107.00 ± 11.00	Cesen et al., 2016 [59]
			BPAF		0.0645	0.215	<LOD	23.02 ± 14.62	53.50 ± 11.00	
			BPE		0.0747	0.249	<LOD	7.14 ± 3.14	12.80 ± 0.70	
			BPF		0.0005 97	0.00199	<LOD	7.96 ± 11.74	31.60 ± 3.90	
			BPS		0.0649	0.216	<LOD	–	302.00 ± 20.00	
			BPZ		0.147	0.489	<LOD	11.28 ± 9.73	28.40 ± 3.00	
	Olive oil (glass)	Spain	BPA	GC-MS	–	0.17	<LOD	–	–	

Fats and oils			BPB		–	–	–	–	0.85	González et al., 2020 [63]
			BPE		–	0.83	<LOD	–	–	
Pastry	Cookies (plastic)	Spain	BPA	GC-MS	–	0.17	<LOD	–	–	González et al., 2020 [63]
			BPB		–	0.17	<LOD	–	–	
			BPE		–	0.17	<LOD	–	–	
Drinking water										
Tap water	Tap water (domestic tap)	France	BPA	LC-MS/MS	-	0.0008	<LOD	0.59 ± 1.10	4.1694	Doumas et al., 2018 [72]
			Clx-BPA (ClBPA, Cl_2BPA, Cl_3BPA, Cl_4BPA)		-	0.0008	<LOD	0.003 ± 0.003	0.0113	
Packaged and unpackaged (unspecified type of container in samples, unspecified non-packaged or mixture of plastic and metal in the container)										
Packaged and canned beverages	22 alcoholic and non-alcoholic beverages packed in different materials, including cans, polyethylene terephthalate (PET) bottles, glass bottles, and carton packs.	Belgium	BPA	LC-MS/MS	1.8-7.1	6.1-23.5	<LOD	–	1.26 ± 0.09	Regueiro and Wenzl, 2015 [78]
			4,4′-BPF		2.9-3.9	9.5-13.1	<LOD	–	1.00 ± 0.08	
			2,4′-BPF		8.0-8.2	26.6-27.2	<LOD	–	0.51 ± 0.06	
			2,2′-BPF		2.4-3.7	8.0-12.2	<LOD	–	0.12 ± 0.02	

LOD. Limit of detection; LOQ. limit of quantification; UHPLC- QqQ/MS. Ultrahigh performance liquid chromatography–triple quadrupole mass spectrometry; GC-MS/MS. Gas chromatography–tandem mass spectrometry; HPLC- FLD. High performance liquid chromatography-fluorescence spectrophotometry; GC. UHPLC-MS/MS. Ultrahigh performance liquid chromatography-tandem mass spectrometry; LC-MS/MS. Liquid chromatography-tandem mass spectrometry; GC-MS. Gas chromatography–mass spectrometry; LC-FD. Liquid chromatography-fluorescence detection; TD-GC-MS. Thermal desorption gas chromatography- mass spectrometry. UHPLC-FD. Ultrahigh performance liquid chromatography-fluorescence detection.

Table S3. Estimated dietary intake of parabens in Spanish children aged 6-9 years.

	Daily intake g day^{-1}*	Mean MetPB ng g^{-1}	Daily intake MetPB ng day^{-1}	Mean EthPB ng g^{-1}	Daily intake EthPB ng day^{-1}	Mean ButPB ng g^{-1}	Daily intake ButPB ng day^{-1}	Mean PropPB ng g^{-1}	Daily intake PropPB ng day^{-1}	Mean ΣPBs ng g^{-}	Daily intake ΣPBs ng day^{-1}
Meat and derivatives	71.96	159.87	11504.03	–	–	9.28	667.43	–	–	99.63	7169.37
Fish and derivatives	40.96	–	–	146.90	6016.73	25.50	1044.43	–	–	172.4	7061.15
Cereals and derivatives	165.93	50.55	8387.81	28.60	4745.63	60.27	10000.16	3.73	619.42	58.383	9687.54
Vegetables and derivatives	132.16	86.67	11454.00	13.90	1837.04	–	–	1.00	132.16	68.65	9072.85
Fruits and derivatives	148.70	48.62	7229.45	47.14	7009.81	43.85	6520.58	67.00	9963.03	74.95	11145.21
Dairy and derivatives	443.14	58.39	25872.61	24.80	10989.82	–	–	–	–	37.39	16569.81
Eggs	18.71	229.90	4301.89	–	–	–	–	–	–	229.90	4301.88
Salty snacks	4.42	7.70	34.05	–	–	–	–	1.4	6.19	4.55	20.12
Pre-cooked	0.76	–	–	–	–	–	–	–	–	–	–
μg kg^{-1} bw day^{-1}			**2.31**		**1.13**		**0.61**		**0.36**		**2.28**

MetPB. Methylparaben; EthPB. Ethylparaben; ButPB. Butylparaben; PropPB. Propylparaben; ΣPBs. Σparabens; bw. Body weight.
(–). Not detected or below of limit quantification. *Data obtained from ENALIA study

Table S4. Estimated dietary intake of bisphenols in Spanish children aged 6-9 years.

	Daily intake g day^{-1}*	Mean BPS ng g^{-1}	Daily intake BPS ng day^{-1}	Mean BPA ng g^{-1}	Daily intake BPA ng day^{-1}	Mean ΣBPs ng g^{-1}	Daily intake ΣBPs ng day^{-1}
Meat and derivatives	71.96	22.37	1609.39	8.67	623.68	17.68	1272.47
Fish and derivatives	40.96	187.80	7691.91	409.00	16751.82	298.40	12221.87
Cereals and derivatives	165.93	2.50	414.83	31.73	5264.16	21.98	3647.66
Vegetables and derivatives	132.16	15.46	2043.21	–	–	15.46	2043.21
Fruits and derivatives	148.70	18.07	2687.05	7.02	1043.14	21.26	3160.81
Dairy and derivatives	443.14	5.25	2326.47	44.09	19536.18	32.99	14619.12
Salty snacks	4.42	132.10	584.15	25.45	112.54	61.00	269.74
Pre-cooked	0.76		–	4.30	3,26	4.30	3.26
μg kg^{-1} bw day^{-1}			**0.58**		**1.45**		**1.25**

BPS. Bisphenol S; BPA. Bisphenol A; ΣBPs. ΣBisphenols; bw. Body weight.
(–). Not detected or below of limit quantification. *Data obtained from ENALIA study
